# Next-generation electrochemical aptasensors for thrombin detection: the rise of aptamer–MOF architectures

**DOI:** 10.1007/s00216-026-06383-8

**Published:** 2026-03-03

**Authors:** Soodabeh Hassanpour, Frank-Michael Matysik

**Affiliations:** 1 Institute of Analytical Chemistry, Chemo- and Biosensors, University of Regensburg, Universitätsstraße 31, 93053 Regensburg, Germany; 2 Department of Chemistry, Faculty of Science, University of Ostrava, 30. Dubna 22, 701 03 Ostrava, Czech Republic

**Keywords:** Thrombin, Metal-organic frameworks, Aptamer-MOF hybrid biosensors, Cancer and coagulation biomarkers, Electrochemical aptasensors, Point-of-care diagnostics

## Abstract

Thrombin is a crucial regulatory enzyme in the blood coagulation cascade that significantly influences cancer metastasis, cardiovascular disease, inflammation, and immunological regulation. Because of its dynamic, concentration-dependent activity, thrombin has emerged as a potential biomarker for the early diagnosis of a wide range of pathological conditions. However, a common drawback of typical thrombin assays is their insufficient specificity, sensitivity, and operational simplicity required for point-of-care or real-time applications. The latest developments in coordination chemistry have resulted in the discovery of metal–organic frameworks (MOFs). These crystalline porous materials have been demonstrated to be robust biosensing platforms due to their extensive surface area, customizable functionality, and biocompatibility. Hence, integrating them with aptamers, synthetic oligonucleotides with remarkable molecular specificity and binding affinity, has led to the emergence of the next generation of electrochemical biosensors called MOF-based aptasensors. The metal–organic framework-based aptasensing strategies outperform current techniques. This review focuses on advancements in MOF–aptamer hybrid platforms and explores their potential to transform from laboratory-scale investigations into clinically viable technologies. Additionally, the study covers a decade of progress from 2015 to 2025, with a focus on innovations in sensor architecture, signal amplification strategies, material synthesis, and clinical applications. Aptamer–MOF complex-based biosensing platforms could revolutionize thrombin diagnosis. This approach leverages molecular diagnostics and supramolecular chemistry to make highly sensitive, portable, and clinically useful devices. This work provides a direct comparative assessment of the clinical performance of MOF–aptamer sensing platforms, classifying them by metal-core chemistry and amplification method. It also outlines commercial and regulatory challenges as well as potential translational prospects. This comprehensive review serves as a design blueprint for developing next-generation biosensors targeting thrombin and other coagulation-related biomarkers.

**Graphical abstract:**

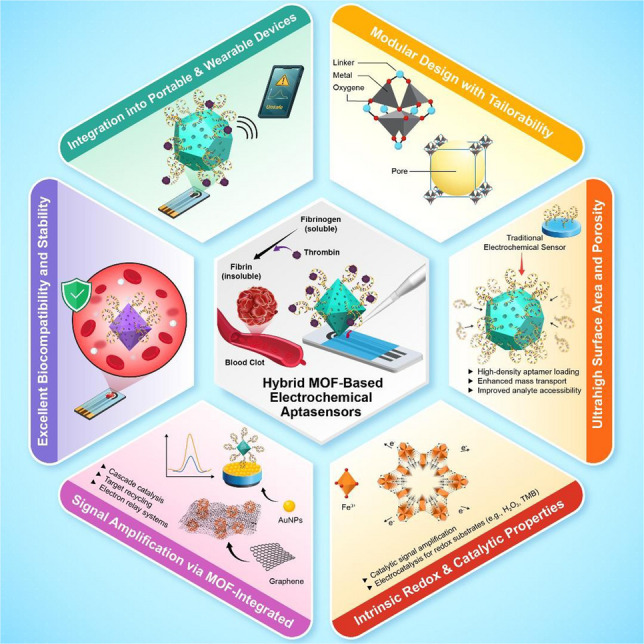

## Thrombin

*Thrombin* (TB) is a Na^+^-activated serine protease from the chymotrypsin family that serves a critical role in the coagulation cascade beyond just being a clotting agent via converting fibrinogen into fibrin after vascular injury [1]. Thrombin is produced from its precursor, prothrombin, and appears in various proteolytic forms, of which α-thrombin is the most physiologically active [2, 3]. Although historically it has long been recognized for its role in hemostasis, research now highlights the extensive biological impacts of thrombin. It has become known as a multifunctional biomarker involved in metastatic cancer, cardiovascular disease, vascular inflammation, and immune regulation [4, 5].

**Fig. 1:**
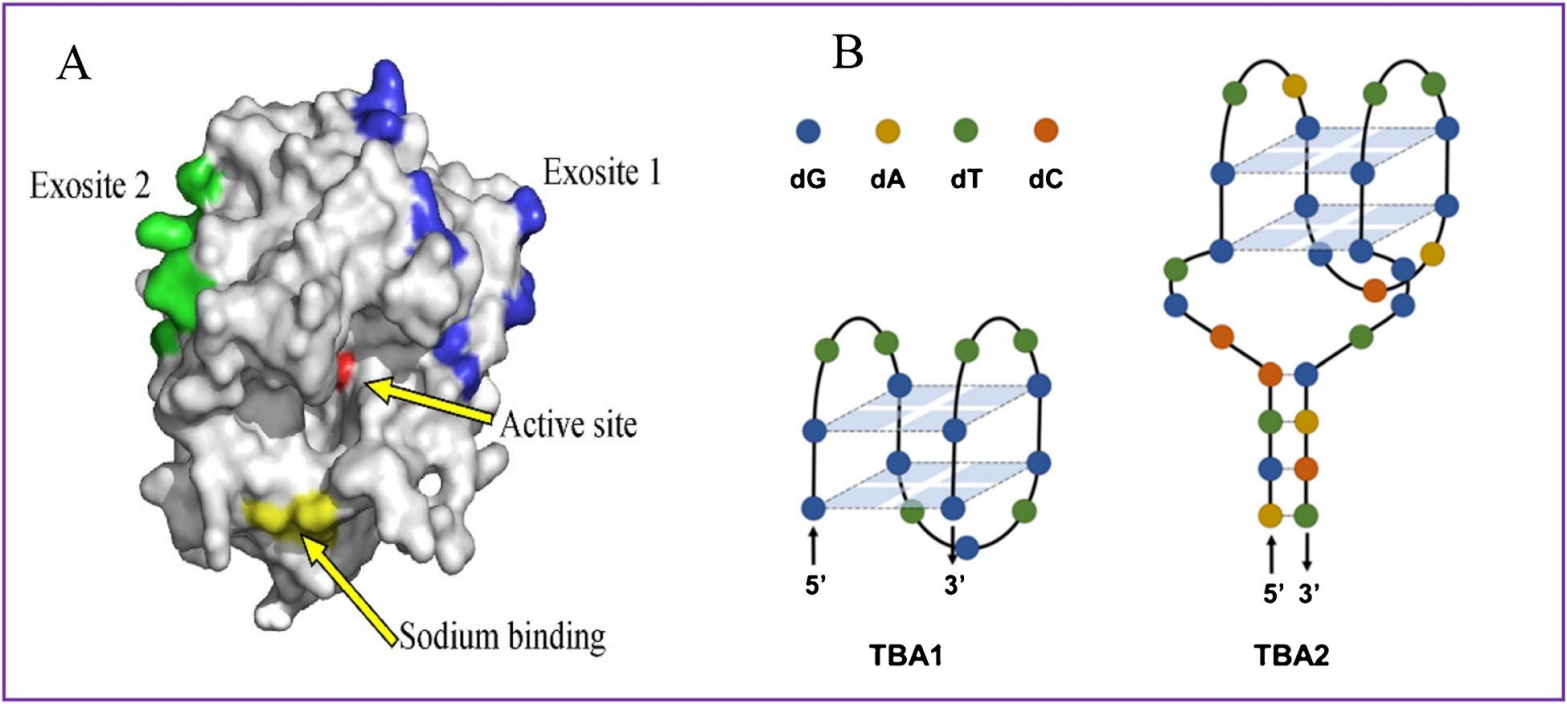
Displayed are the G-quadruplex structures formed by TBA1 and TBA2, with their deoxynucleotide sequences, highlighting guanine (dG), adenine (dA), thymine (dT), and cytosine (dC) bases. (**A**) presents the 3D protein structure of α-thrombin with exosite I and II binding sites. Adapted from [8], with permission from *Journal of Thrombosis and Haemostasis*. (**B**) provides a schematic of the aptamers’ nucleotide pairing and motifs, with the 5′ and 3′ ends marked. Adapted from [9], with permission from *MDPI Chemosensors*, 2022, and *Molecular Therapy Nucleic Acids*, 2022

Thrombin contains three functionally unique domains on its surface: the active site, the fibrinogen-recognition exosite (exosite I), and the heparin-binding exosite (exosite II) (Fig. 1(A)). These structural properties enable it to interact with a variety of cofactors and substrates, adjusting its biological effects accordingly. Thrombin’s concentration-dependent activation of protease-activated receptors (PARs) and its extracellular interactions contribute to its broad pathophysiological significance [4, 5]. However, dysregulation in the activity of thrombin and abnormal thrombin levels, and its overexpression are associated with several life-threatening disorders, including stroke, liver dysfunction, deep vein thrombosis, cardiovascular disease, pulmonary embolism, and various cancers [4, 6, 7].

This profound pathophysiological importance has spurred efforts to develop highly sensitive technologies for thrombin detection. This profound pathophysiological importance has spurred efforts to develop highly sensitive technologies for thrombin detection. Conventional approaches, such as ELISA, chromogenic substrate assays, and mass spectrometry, face considerable downsides, including laborious procedures, high cost, time-consuming nature, and incompatibility with rapid and point-of-care (POC) applications [4, 10]. To address these drawbacks, among emerging biosensing platforms, electrochemical aptasensors, which combine high-affinity aptamers with electrochemical interfaces, have attracted significant attention for their exceptional sensitivity, label-free detection, low cost, and ease of miniaturization. Their strong analytical performance, specificity, and real-time adaptability make them highly promising for early thrombin detection and future clinical applications [4, 11–13]. Notably, studies have shown that the design of functional materials, which plays a crucial role in enhancing sensor sensitivity, operational stability, and biocompatibility, strongly influences the practical performance of these biosensors [14–16].

Following the first report in the early 1990 s, aptamers rapidly emerged as promising alternatives to antibodies due to their cost efficiency, chemical stability, and flexibility [17]. Aptamers are short, synthetic single-stranded RNA or DNA oligonucleotides that can fold into unique 3D structures, allowing them to bind targets such as proteins, small molecules, nucleotides, peptides, and cells with high specificity and sensitivity. They are usually synthesized through the Systematic Evolution of Ligands by Exponential Enrichment (SELEX) method [18–20]. Aptamers are attractive biorecognition components due to their high thermal stability, low immunogenicity, small size, simplicity of chemical modification, and cost-effective synthesis. Often called “chemical antibodies,” aptamers offer advantages over traditional antibodies, including faster production, greater reproducibility, and compatibility with nanomaterials [15, 21–24].

Since Bock et al. first reported the thrombin-binding aptamer (TBA) in 1992, many aptamer sequences have been developed to target human α-thrombin [25]. Two have stood out as the most widely used in biosensor applications. The first and most well-known is the 15-mer aptamer (5′-GGT TGG TGT GGT TGG-3′), named TBA1. It forms a stable antiparallel G-quadruplex structure with a chair-like conformation and binds specifically to exosite I with a dissociation constant (Kd) of ~ 100 nM. The second is the 29-mer aptamer (5′-AGT CCG TGG TAG GGC AGG TTG GGG TGA CT-3′), or TBA2, discovered by Tasset et al. in 1997. TBA2 binds to exosite II of thrombin with a much higher affinity (Kd ~ 0.5 nM) and also forms a G-quadruplex embedded within a stem-loop structure (Fig. 1(B)) [9, 25–29].

These short oligonucleotides are simple and affordable to synthesize, and can be modified with capture or reporter groups. These aptamers’ non-overlapping binding sites on thrombin allow dual-site binding in sandwich-format aptasensors, enhancing specificity and signal amplification. Both aptamers form four-stranded DNA helices, G-quadruplex structures stabilized by cations like K⁺ and guanine tetrads. This allows hemin to bind and create catalytically active DNAzymes capable of catalyzing H₂O₂-mediated oxidation reactions, which are beneficial for signal generation. These DNAzymes play a critical role in catalytic amplification in thrombin aptasensors. The modular design, high affinity, and structural stability of these aptamers support their application in clinical diagnostics and in next-generation metal–organic framework (MOF)-based biosensors [27, 30, 31].

In particular, the use of MOFs as electrode modifiers improves electron-transfer efficiency, aptamer immobilization, and target accessibility. According to their inherent redox activity, high porosity, and tailored surface chemistry, MOF–aptamer platforms are especially well-suited for real-time thrombin-sensing applications. Their potential as next-generation bioreceptors for POC diagnostics and biosensors has been reinforced by recent advancements in aptamer chemical modifications and nanomaterial conjugation, which have resulted in further improvements in their sensing capabilities and structural stability [15, 20, 32, 33]. For this purpose, extensive investigation has been conducted on functionalizing aptamers with MOFs to enhance their binding capacity, durability, and multifunctionality. Metal–organic frameworks provide a suitable matrix for aptamer anchoring because of their tunable pore structures, large surface areas, and functional groups. These features also lead to effective signal amplification in electrochemical biosensors [16, 20].

Hence, this article provides an in-depth study of the development of electrochemical aptasensors based on metal–organic frameworks for thrombin detection, spanning studies published from 2015 to 2025 and bridging material design with translational diagnostic applications. This time frame was selected to showcase the concurrent advancement in supramolecular chemistry and electrochemical aptasensing, alongside the rising need for point-of-care biomedical diagnostics. This review aims to provide a framework for future research developing clinically versatile thrombin diagnostics by highlighting key innovations, remaining limitations, and translational pathways.

## Metal–organic frameworks

The concept of metal–organic frameworks was first introduced by O.M. Yaghi and colleagues in 1995, marking the beginning of a versatile class of porous coordination polymers. These materials exhibit 1D, 2D, or 3D inorganic–organic crystalline architectures [34, 35]. Metal–organic frameworks represent a unique category of crystalline porous materials that have attracted considerable attention in recent decades due to their tunable structures and multifunctional properties. MOFs built from metal ions or clusters linked by organic linkers combine the advantages of inorganic and organic components, resulting in high surface areas, tunable porosity, flexible polymorphism structure, superior catalytic activity, biodegradability, biocompatibility, structural diversity, and configurable chemical functionalities [15, 33]. Their remarkable physicochemical stability and structural versatility make them attractive for a range of applications, including sensing technologies, gas storage, catalysis, and drug delivery. MOFs have emerged as powerful biosensing platforms for electrode modification, with high adsorption capacity, efficient electron-transfer, and abundant binding sites for biorecognition elements such as aptamers [35, 36].

It is worth mentioning that the performance of MOF-based aptasensors is influenced not only by the porosity and surface chemistry of MOFs, but also by the type of central metal core. The metal core of MOFs strongly influences their electrical properties, stability, and interactions with biomolecules [37, 38]. Therefore, the following section investigates how different metal cores of MOFs affect sensor performance, focusing on optimizing sensitivity, selectivity, and stability in thrombin detection.

### Metal core effect on sensor performance

The central metal ion or cluster in metal–organic frameworks is a critical component of the physicochemical features and general performance of MOF-based electrochemical aptasensors. In addition to their role as coordination nodes for organic linkers, they also affect important factors like redox behavior, catalytic activity, electrical conductivity, and structural stability. These properties are directly related to the aptamers’ immobilization, electron-transfer proficiency at the electrode interface, electrochemical signal amplification, and the selectivity and sensitivity in biosensing applications, particularly for detecting thrombin. The performance of biosensing platforms can be improved by tailoring MOF architectures through careful selection and engineering of metal cores, which can vary from transition metals to noble metals and even mixed-metal systems [20, 33, 39, 40].

**Fig. 2:**
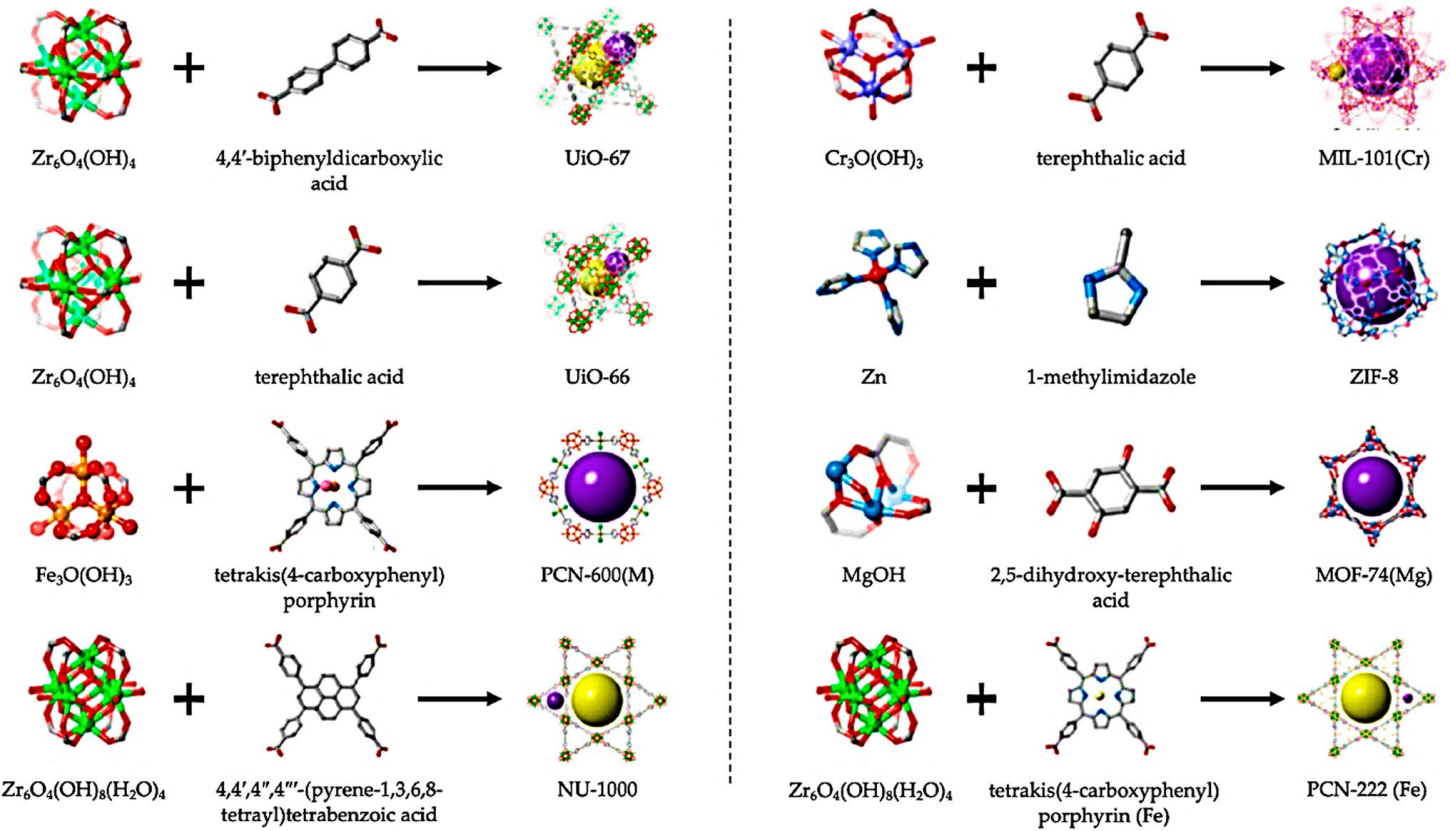
Examples of diverse metal–organic framework (MOF) architectures highlighting how different metallic clusters and organic linkers combine to form unique porous frameworks. Each MOF shown represents a distinct assembly of metal nodes and organic molecules, resulting in tailored structural and functional properties relevant for applications such as gas storage, separation, catalysis, and biosensing. Adapted from [46, 47], with permission from *MDPI Separations*, 2019, and *American Chemical Society*, 2017

The broad spectrum of structures in MOFs originates from the coordination of different metal ions (e.g., Cu^2^⁺, Zn^2^⁺, Cd^2^⁺, Fe^3^⁺, Ln^3^⁺, and Al^3^⁺) with organic linkers, including phosphonates, carboxylates, sulfonates, and nitrogen-containing heterocycles, which facilitate framework formation (Fig. 2) [20, 24, 41]. For instance, copper-containing metal–organic frameworks (Cu-MOFs), such as Cu-TCPP and Cu-BTC, are widely used in electrochemical sensors due to their high electrocatalytic activity and ability to mediate electron-transfer reactions. The redox-active Cu(II)/Cu(I) couple enables efficient electron cycling and boosts signal generation during target recognition events. Their heightened electrochemical activity makes them appropriate platforms for signal transduction in thrombin aptasensors [42, 43]. Iron-based MOFs (Fe-MOFs) have inherent peroxidase-like catalytic activity, such as MIL-88 and MIL-101 from the MIL family. This characteristic enables the catalytic decomposition of H₂O₂ (hydrogen peroxide), a typical signaling molecule in biosensing procedures, thereby simplifying signal amplification in amperometric or colorimetric experiments. These Fe-MOFs serve dual functions as structural platforms and catalytic amplifiers, thereby improving the overall sensitivity of the detection system [44, 45].

Nickel-containing metal–organic frameworks (Ni-MOFs) are known for their high conductivity and stability under electrochemical environments. Their potential to facilitate rapid electron-transfer kinetics increases sensitivity and yields ultralow detection limits. For example, detection of thrombin employing Ni-MOF-based sensors has achieved sensitivities as low as 0.016 pM, emphasizing their promise for early-stage clinical diagnostics [48, 49]. Zirconium metal–organic frameworks (Zr-MOFs) possess structural and chemical durability (e.g., Uio-66), which arises from the stable and robust coordination between the carboxylate ligand and the Zr^4^⁺ ion. This interaction results in the construction of sturdy frameworks and the maintenance of integrity under harsh conditions. These properties make Zr-MOFs well-suited for biosensors that require durability, biocompatibility, and extended storage in clinical or point-of-care applications [50–52].

In addition, various metal ions can be incorporated into a single MOF structure, forming bimetallic MOFs that leverage synergistic effects and surpass the performance of individual metal components, such as Fe/Zn–MOFs or Ni/Co–MOFs. The combination of the catalytic oxidation ability of Co^2^⁺ with the high conductivity of Ni^2^⁺ centers in Ni/Co–MOFs enhances electron-transfer kinetics, conductivity, and electrocatalytic activity. These conjugations affect surface charge and aptamer orientation while boosting detection selectivity and sensitivity of MOF-based aptasensors by amplifying the electrochemical signal and analyte binding. The choice of metal core influences both the electrical and catalytic characteristics of metal–organic frameworks and their compatibility with aptamer immobilization and target recognition [53–57]. The properties of metal cores significantly shape sensor performance and provide the foundation for investigating the underlying mechanistic principles in the next section, which are governed by the framework’s functionality and metal–ligand coordination.

### Mechanistic insights: metal-ligand coordination and functionality

Beyond the role of metal nodes, the interactions among surface chemistry, framework structure, and nucleic acid recognition dynamics determine the mechanistic performance of MOF-aptasensors [58]. The strategies for aptamer immobilization, ranging from non-covalent π-π stacking, electrostatic adsorption, and hydrogen bonding to covalent binding via amino, thiol, or carboxyl groups, directly control both the stability of the biorecognition layer and the efficiency of target-induced conformational switching [24, 35, 59]. Meanwhile, MOFs’ electron-transport pathways and porous nature are crucial for transducing aptamer conformational changes into measurable electrochemical signals. Hence, insight into these mechanistic principles is paramount for rational design, as it allows integration of MOF architectures and surface chemistries with aptamer properties to acquire improved selectivity, robustness, and signal amplification in thrombin detection [58, 60].

Coordination chemistry within metal–organic frameworks enables controlled function and structure, in which the coordination environment of the metal centers controls framework topology, stability, and electrochemical properties. Tetrahedral and octahedral structures within ZIF-8 and Fe-MOF, as well as trigonal bipyramidal structures composed of specific ligand field environments and coordination numbers of the metal cores, yield different physicochemical attributes and structural motifs. Organic linkers play a pivotal role in adjusting the morphology, pore size, and chemical reactivity of MOFs, as well as introducing functional groups (-OH, -SO_3_H, and -NH_2_), which can be used for covalent attachment of biological probes or post-synthetic modification. Moreover, the metal cores in MOFs contribute to the framework’s structural rigidity and can confer redox or catalytic activity depending on their coordination environment and oxidation state [61–63]. This composition of modular inorganic and organic elements provides a high degree of tunability for MOFs, surpassing that of conventional porous materials such as mesoporous silica and zeolites. Subsequently, it enables precise modulation of pore sizes ranging from sub-nanometer to several nanometers, along with tailored surface chemistry [33, 35].

The structural features of MOFs enable efficient immobilization of guanine-rich aptamers on their surfaces. Aptamer functional groups can strongly bind to exposed metal sites of MOFs via metal–amine or metal–phosphate interactions, thereby improving immobilization efficacy while maintaining the natural aptamer conformation. Effective coordination of DNA backbone phosphate groups with metal ions such as Fe^3^⁺, Zr^4^⁺, or Ce^4^⁺ provides firm aptamer anchoring to the MOF surface while preserving recognition potential [35, 59, 60, 64].

MOFs can function as inherent signal transducers via redox-active metal ions within the framework, beyond serving as a platform for aptamer immobilization. In Ce–MOFs and Cu–MOFs, cerium and copper act as catalytic centers, undergo reversible redox cycling, directly mediate electron transfer, and amplify the electrochemical signal without additional mediators. This intrinsic catalytic activity streamlines sensor design and enriches sensitivity and stability. Moreover, their porosity and interconnected channels provide large surface areas, facilitating rapid analyte diffusion and reducing the steric barrier at the electrode interface, thereby increasing local analyte concentration near immobilized aptamers, accelerating binding kinetics, and enhancing detection efficacy [40, 42].

The structural porosity, intrinsic redox activity, and coordination chemistry all together increase signal transduction, stability, and specificity in MOF-based aptasensors. These properties highlight their potential as promising candidates for sensitive, real-time, and point-of-care thrombin detection [44, 58]. Based on this chemical and structural foundation, the next essential part is to explore how these MOF platforms are combined with different signal amplification strategies to extend detection limits and reinforce analytical performance.

### Signal amplification strategies and challenges in MOF-based electrochemical aptasensors

The chemical and structural characteristics of MOFs provide a basis for aptamer immobilization; however, achieving practical clinical detection ranges often requires additional signal amplification steps. To convert the molecular recognition event between thrombin and its aptamer into a measurable electrochemical response, a signal amplification strategy is necessary to achieve high selectivity and sensitivity [65]. In MOF-aptasensors, amplification can be achieved through various complementary methods, such as nanomaterial-assisted electron-transfer enhancement, catalytic amplification using integrated nanozymes or redox-active MOF nodes, and biomolecular amplification mechanisms, including rolling circle amplification (RCA), DNA walkers, and hybridization chain reaction (HCR). The combination of amplification approaches with the selective binding affinity of aptamers enables MOF-based electrochemical biosensors to detect thrombin at concentrations significantly below physiological levels. Hence, this helps to bridge the transition from fundamental nanomaterial design to applied biomedical applications [12, 15, 60].

Among the widely utilized strategies is catalytic redox cycling, in which redox-active metal-composed MOFs, such as Cu^2^⁺, Fe^3^⁺, or cerium (Ce^4^⁺), partake in multi-electron-transfer reactions. Based on these reactions, amplification of the generated electrochemical signal during thrombin-aptamer interaction is achieved through constant electron-exchange methods, thereby enhancing the current output per recognition event. Integration of conductive nanomaterials, such as carbon nanotubes (CNTs), graphene oxide (GO), and gold nanoparticles (AuNPs) with metal–organic frameworks has become proficient in addressing the low conductivity of pristine MOFs. Combining these electroactive compounds increases the electrochemical surface area and signal intensity, and improves electron-transfer kinetics. Such hybridization offers advantages, including the preservation of aptamers’ molecular recognition capabilities, optimization of sensor conductivity, and enhanced signal amplification [42, 60, 65, 66].

DNAzyme amplification has been shown as another potent approach, especially suitable for thrombin aptasensing. In the presence of potassium ions and hemin, TBA15 assembles into hemin-DNAzyme complexes, enabling the catalysis of H₂O₂ reduction to water and thereby amplifying electrochemical signals in comparison to those generated by direct target binding [12, 67]. Enzyme-free target recycling methods have been demonstrated to offer an additional increment in sensitivity. In these cyclic amplification approaches, a single thrombin molecule initiates multiple signal-generating events. For instance, a trigger strand that begins after hybridization and cleavage cycles is released by the initiation of target-induced duplex DNA cleavage via Exonuclease III (Exo III)–assisted recycling. Likewise, target-triggered strand displacement reactions exploit the thermodynamics of nucleic acid interactions to amplify signals without enzyme involvement [68–71].

To enhance signal transduction and specificity, the dual-aptamer configurations are also widely employed. For the simultaneous identification of exosite I and exosite II thrombin epitopes, TBA15 and TBA29 can be used in combination. The benefit of dual-site binding for sandwich-type sensor platforms is the ability to achieve precise and measurable results with reduced background noise [31, 72]. Finally, calcination results in the formation of MOF-derived nanocomposites, demonstrating a new category of materials with enriched electrochemical performances. Under inert conditions, the thermal decomposition of MOFs produces porous carbon frameworks that contain metal oxides or metal nanoparticles. These hybrid networks leverage the parent MOF’s large surface area, resulting in higher stability, conductivity, and catalytic performance in comparison to the pristine MOFs. Ultra-sensitive detection of thrombin with fast response times and high reproducibility has been made possible by their usage in aptasensor design [73, 74].

In spite of the considerable progress in signal amplification strategies, the development of amplified MOF-aptamer platforms with reliable repeatability, stability, and clinical applicability remains a key challenge [75]. Regardless of the efficiency of enzymatic amplification designs, such as those based on DNAzymes and Exo III, they also exhibit low thermal stability and a short lifespan in ambient environments [65]. Hybrid nanomaterials, such as MOF-CNT or MOF-AuNP composites, are prone to aggregation under physiological conditions or storage, which adversely affects signal consistency [76]. Moreover, due to the multistage incubation and washing steps in amplification strategies, these strategies are complicated and impractical for POC diagnosis. The limited conductivity of many pristine MOFs further exacerbates these issues, which require tailored engineering with redox-active metals or a combination with conductive materials to achieve efficient electron transfer. The development of durable nanozyme-based amplification procedures with wash-free or one-step protocols, and the fabrication of stimuli-responsive MOFs capable of dynamically modulating signals, should be the focus of ongoing research. The usage of ternary metal nodes could pave the way for multifunctional platforms capable of aptamer anchoring, redox cycling, and catalytic amplification. Thus, these observations demonstrate the effectiveness of amplification strategies in improving the specificity and sensitivity of MOF-based electrochemical aptasensors [65, 75].

However, addressing these shortcomings with simplified methods will connect fundamental research with practical applications. Consequently, these insights reveal the need for material advancements and technological innovations, as well as opportunities for a comparative evaluation of MOF–aptasensors for thrombin diagnosis.

## Comparative evaluation and distinct advantages of MOF-aptamer electrochemical thrombin sensors

A comprehensive study of MOF-based electrochemical aptasensors highlights their merits, which stand out from those of conventional biosensing platforms [60]. Over the last decade, various MOF-based configurations and detection platforms have appeared, with label-free, dual-readout, and sandwich-type architectures attracting attention for their adaptability and analytical performance. Label-free sensors offer low cost, simplicity, and minimal signal interference for thrombin diagnosis. These biosensors typically measure alterations in current, impedance, or potential shifts during target binding. Although integrating MOFs into these systems increases the electrode surface area and promotes stronger analyte interaction, in complex media, label-free systems may face reduced selectivity or inadequate signal-to-noise ratios unless modified with signal amplification methods [12, 35, 77, 78].

In dual-readout aptasensing procedures, to enhance accuracy and precision, the electrochemical detection mode is combined with colorimetric or optical outputs. The suitability of MOFs for these platforms supports the use of multi-signal mechanisms within a single system. For instance, redox-active MOFs, such as Ce-MOFs and Cu-MOFs, can catalyze chromogenic reactions, leading to the built-in amplification of both detection modes. Although they improve sensitivity and reliability, their construction and signal calibration are more complicated [79–81]. Sandwich-type platforms commonly use dual aptamers, such as TBA29 and TBA15, to specifically target two thrombin epitopes, thereby ensuring high detection accuracy. By integrating these configurations with MOF nanohybrids, such as MOF-CNT or MOF-AuNP composites, ultralow detection limits in the femto- to picomolar range are achieved, and compatibility with POC designs is ensured due to their modular design [31, 60].

Aptamer-MOF hybrids, because of their specific functional and structural attributes, outperform conventional nanomaterials, including graphene oxide, carbon nanotubes, and metal nanoparticles. They enable the development of diverse, multifunctional biosensing platforms, in which their 3D porous structure permits the co-immobilization of signal and recognition elements, resulting in the improvement of both stability and accessibility. Furthermore, the chemical flexibility of MOFs helps hybridization with nanocomposites and polymers, making them feasible for integration into wearable or portable biosensing devices [20, 35, 82].

Thus, alongside the unique strengths of different configurations of MOF–aptamer hybrid thrombin sensors, they also demonstrate excellent multifunctionality and amplification capacity, leading to high sensitivity and durability. Their optimal analytical performance relies on the stability, effectiveness, and orientation of aptamer immobilization within the MOF matrix, a key factor discussed in the following section.

## Aptamer immobilization on MOFs

The crucial factor in the fabrication of high-performance electrochemical aptaensors, particularly for thrombin detection, is the stable and efficient immobilization of aptamers onto MOFs. It is necessary to have proper density, stability, and orientation for aptamers to perform effectively in sensing platforms. The selected immobilization method exhibits both the strength of MOF–aptamer interactions and the significant effect of MOF physicochemical characteristics, such as biocompatibility, dispersibility, electron transfer at the electrode interface, and general sensing functionality. The classification of aptamer anchoring strategies encompasses both noncovalent and covalent procedures, each presenting distinct benefits based on the application necessities [24, 35].

### Covalent immobilization

One of the most widely used approaches is covalent immobilization due to its excellent robustness, stability, and reproducibility under various physiological and electrochemical conditions. Particularly, the formation of the amide bond between the primary amine (–NH₂) groups on the MOF organic linkers and the carboxyl (–COOH) groups on aptamers results in the achievement of covalent binding. Carbodiimide coupling agents, such as EDC/NHS, typically ease the formation of covalent bonds by activating carboxyl groups [24]. Moreover, incorporating metal elements into MOFs enables them to bind nucleic acids via an alternative covalent approach. The immobilization of thiol-functionalized aptamers onto MOFs with soft-metal sites or AuNPs is achieved via strong Au–S interactions, thereby enhancing electron-transfer efficiency and the structural integrity of the electrode. Further, MOFs with Fe^3^⁺, Zr^4^⁺, or Ce^4^⁺ metal nodes can form stable phosphate-metal bonds by direct coordination to phosphate groups on aptamer backbones. These covalent procedures provide long-lasting, robust immobilization with great signal stability, making them promising for clinical biosensing applications [35, 59, 62].

### Non-covalent immobilization

Another strategy for integrating MOFs with aptamers for biosensing applications is non-covalent binding, in which the primary mechanisms rely on weak interactions, including van der Waals forces, π-π interactions, hydrogen bonding, and electrostatic forces. Non-covalent strategies provide a simpler, less chemically invasive alternative for anchoring aptamers to MOFs. Although these bonds are less permanent than covalent linkages, they help preserve the intrinsic structure, porosity, and redox activity of MOFs, an advantage when maintaining functional integrity is critical. Sometimes, numerous non-covalent interactions bind aptamers, DNA, and RNA to MOFs. For instance, achieving both reversible and stable immobilization can be accomplished by electrostatic attraction between positively charged linkers or metal nodes in MOFs, such as UiO-66 and MIL-101 derivatives, and negatively charged DNA aptamers. Although non-covalent immobilization offers limited robustness under intense electrochemical environments, it supplies flexibility and reversibility, which is profitable for the fabrication of reusable or regenerable sensing platforms [35, 59, 83].

### Hybrid immobilization strategies

In order to enhance signal stability and immobilization robustness, hybrid immobilization strategies have emerged that combine non-covalent and covalent interactions. While non-covalent linkages in these mixed-mode systems preserve the electroactive sites and intrinsic porosity of MOFs, covalent binding simultaneously leverages the strong, specific immobilization of the aptamer. The immobilization efficacy, reusability, and signal stability are improved by hybrid approaches, which, in turn, result in the enhancement of sensitivity, reproducibility, and biocompatibility of sensors. Hence, dual-mode immobilization provides a well-balanced strategy for advancing high-performance, durable thrombin aptasensors suitable for prolonged and reusable clinical applications. By performing both as electroactive signal amplifiers and aptamer carriers, MOF-aptamer hybrid platforms have emerged as a strong candidate in the development of next-generation thrombin biosensors [35, 59].

In conclusion, the critical parameter in the performance of hybrid MOF–aptamer sensors is the immobilization of the aptamer, which directly influences sensitivity, stability, and reusability. The following section explores hybrid MOF-based electrochemical aptasensors for the detection of thrombin.

## Hybrid MOF-based electrochemical aptasensors for thrombin detection

In recent years, promising potential for the development of next-generation diagnostics for thrombin determination has been demonstrated by electrochemical biosensors based on hybrid MOF-aptamer complexes. Alongside increased electron-transfer and biocompatibility, the collaborative interaction of aptamers and MOFs enables highly sensitive thrombin detection suitable for early diagnosis of coagulation-associated disorders. These hybrid systems have been used in different configurations, involving label-free [77], dual-readout, and sandwich-type formats [78], achieving detection limits down to femtomolar levels. In addition to configuration-based designs, current research has highlighted that the chemical composition of MOF metal centers and related amplification strategies significantly affect sensing performance [24, 84]. Therefore, this section categorizes MOF-aptamer thrombin biosensors based on their metal node composition and primary signal amplification processes:

### Monometallic MOFs with intrinsic redox activity

Monometallic MOFs with intrinsic redox activity represent one of the most extensively studied classes of hybrid MOF–aptamer electrochemical biosensors for the determination of thrombin.

These platforms are composed of a single metal center like Ce^3^⁺/Ce^4^⁺, Fe^3^⁺/Fe^2^⁺, Ni^2^⁺, Ti^4^⁺, Cu^2^⁺, or Zr^4^⁺ with inherent catalytic or electrochemical activity, which causes the generation of electrochemical signals without the requirement of external redox mediators. By utilizing their native redox activity, these MOFs facilitate sensor architecture and supply stable signal transduction. Furthermore, integration of monometallic MOFs with conductive nanomaterials such as AuNPs and PtNPs facilitates aptamer immobilization, improves conductivity, and amplifies the electrochemical signal, which consequently boosts the sensitivity and reliability of the biosensor [35, 85, 86] 

**Fig. 3:**
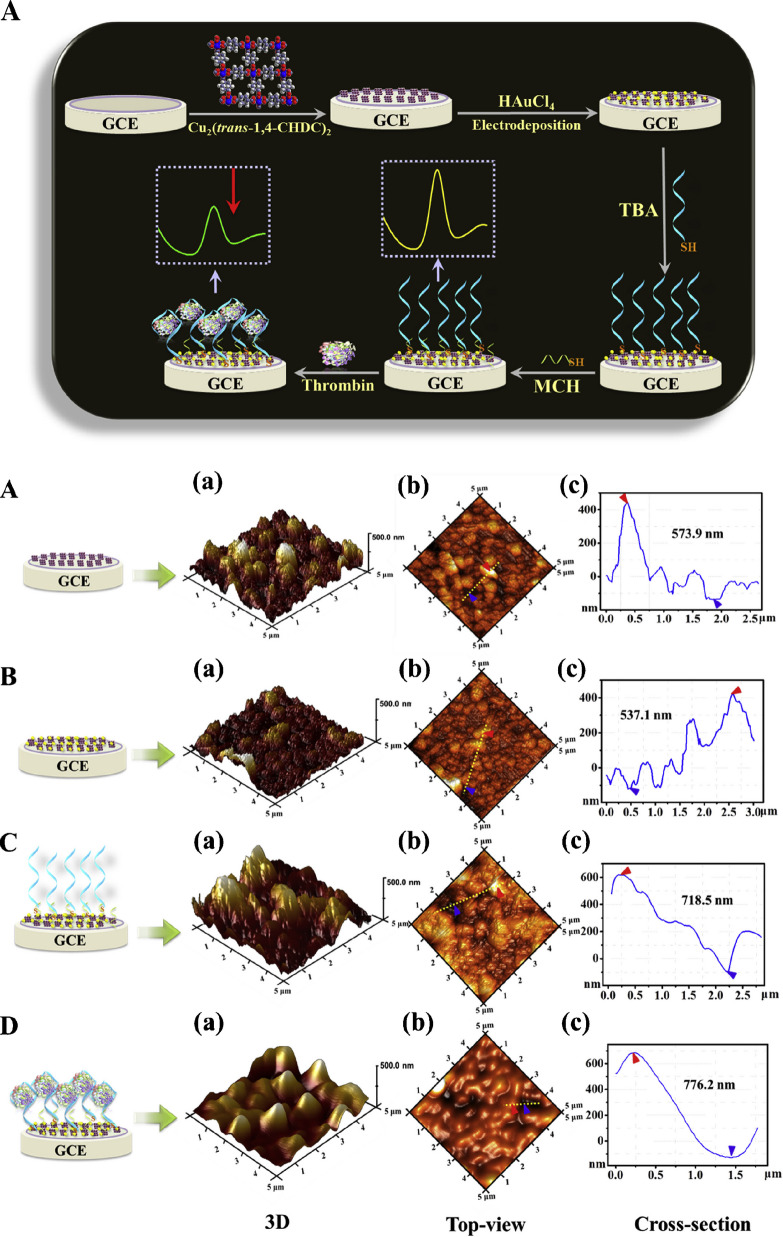

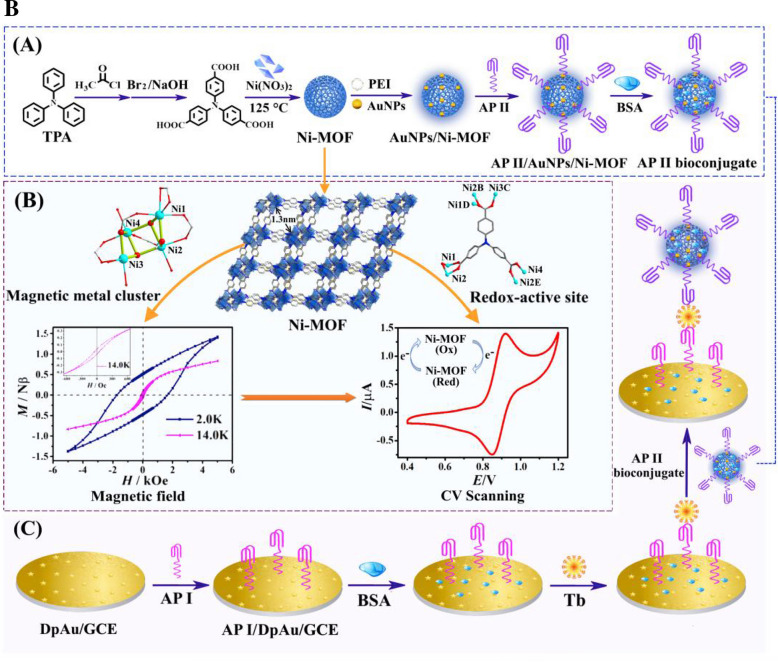
(**A**) A schema of the development process of Cu_2_(CHDC)_2_ electrochemical thrombin aptasensor as well as (a) 3D, (b) top-viewed, and (c) cross-sectional AFM images of each electrode modification step. Adapted from [87], with permission from the *Journal of Electrochimica Acta*, 2020. (**B**) Schematic depiction of (a) the synthesis process of the signal probe AP II/AuNPs/Ni-MOF, (b) structural effects on the electrochemical and magnetic properties of Ni-MOF, and (c) the sandwich-type electrochemical aptasensor construction process for detection of thrombin. Adapted from [88], with permission from the *Journal of Analytica Chimica Acta*, 2019

One representative example is a label-free electrochemical aptasensor developed by Tanaka H. et al. [87] for the detection of thrombin using a novel pillar-layer flexible MOF Cu_2_(CHDC)_2_ (Cu_2_(trans-1,4-cyclohexane dicarboxylic acid)_2_), where the Cu(II) paddle-wheel nodes supply innate redox activity for direct, label-free electrochemical detection without the need for external mediators. These Cu(II) centers also provide abundant, electrochemically accessible active sites for aptamer-thrombin interactions, thereby enhancing the signal response. A simple one-step hydrothermal technique was employed to synthesize Cu_2_(CHDC)_2_, resulting in a flower-like nanocluster-assembled morphology with robust mechanical and thermal stability, a high surface area, and good electroactivity. The mechanical hydrophobicity, robustness, and partial flexibility of MOF were related to hydrophobic -CHDC linkers and the rigid Cu_2_(COO)_4_ core, and the hydrophobic -CHDC linkers, which inhibited average elasticity and resistance to structural collapse in aqueous conditions. For electrode preparation, Tanaka H. and colleagues deposited the Cu₂(CHDC)₂ MOF onto a glassy carbon electrode (GCE) and subsequently electrodeposited AuNPs to form the composite AuNPs/Cu₂(CHDC)₂/GCE which was used for immobilization of the thrombin aptamer. The developed platform utilized the synergistic effect of the Cu-Au interface, where the hierarchical porous structure of the MOF facilitated fast electron transfer, and AuNPs enhanced conductivity as well as providing Au–S binding sites for covalently immobilizing the thiolated-TBA (Fig. 3(A)). During the sensing mechanism, the immobilized aptamer recognized thrombin by generating a “signal-off” response through electrostatic effects and steric hindrance, which declines the faradaic current associated with the Cu(II)/Cu(I) redox couple. The proposed MOF-based aptasensor exhibited high sensitivity with a broad linear range and a detection limit (LOD) of 0.01 fM. However, the irreversible interaction of the aptamer with target thrombin, along with the unclear signal-off mechanism, restricts the reusability of the aptasensor and limits opportunities for further optimization and extension of its analytical performance [87].

Similarly, Chen S. et al. [88] developed a highly stable electrochemical aptasensing platform based on an electroactive Ni-MOF (nickel-based MOF) incorporated with the redox-active ligands H3TCA (4,4′,4″-tricarboxytriphenylamine) and magnetic Ni_4_O_4_ clusters as electronic transport nodes for the sensitive detection of thrombin. An increment in the intrinsic electroactivity of MOF due to the merging of the conjugated triphenylamine structure with the Ni^2^⁺/Ni^3^⁺redox pair resulted in the reduction of the necessity of post-synthetic modification or external mediators for signal generation. In the meantime, the magnetic properties of NiO₄ clusters enable their assembly into biosensing platforms. The designed sandwich-type aptasensor leverages the high aptamer-loading capacity of the Ni-MOF and its multiple electroactive sites to enhance selectivity and sensitivity. First, AuNPs were electrodeposited onto the GCE surface, forming a conductive gold nanoparticle film (DpAu). Next, the Ni-MOF surface was functionalized with polyethyleneimine to introduce abundant amine groups, which enabled the anchoring of additional AuNPs via amino-Au affinity interactions, producing the AuNPs/Ni-MOF hybrid (Fig. 3(B)) and improving aptamer immobilization. In the final sensing configuration, the capture aptamer (AP I) was immobilized onto the DpAu/GCE. In contrast, the detection aptamer (AP II) was bioconjugated to AuNPs/Ni-MOF to form the signal probe (AP II/AuNPs/Ni-MOF). Through the binding of thrombin, the electroactive Ni-MOF probe is positioned closely to the electrode in the sandwich configuration, resulting in an intense current response due to the synergy between the conductive Au network and the Ni redox centers. The hybrid Ni-Au-aptamer interface demonstrated analytical performance, including reproducibility, stability, and selectivity against interfering proteins, with a linear range of 0.05 pM to 50 nM and an LOD of 0.016 pM. The platform illustrates the mechanism of combining conductive nanostructures with inherently electroactive MOF, and the optimization of biofunctionalization can lead to a next-generation platform for thrombin detection [88].

**Fig. 4:**
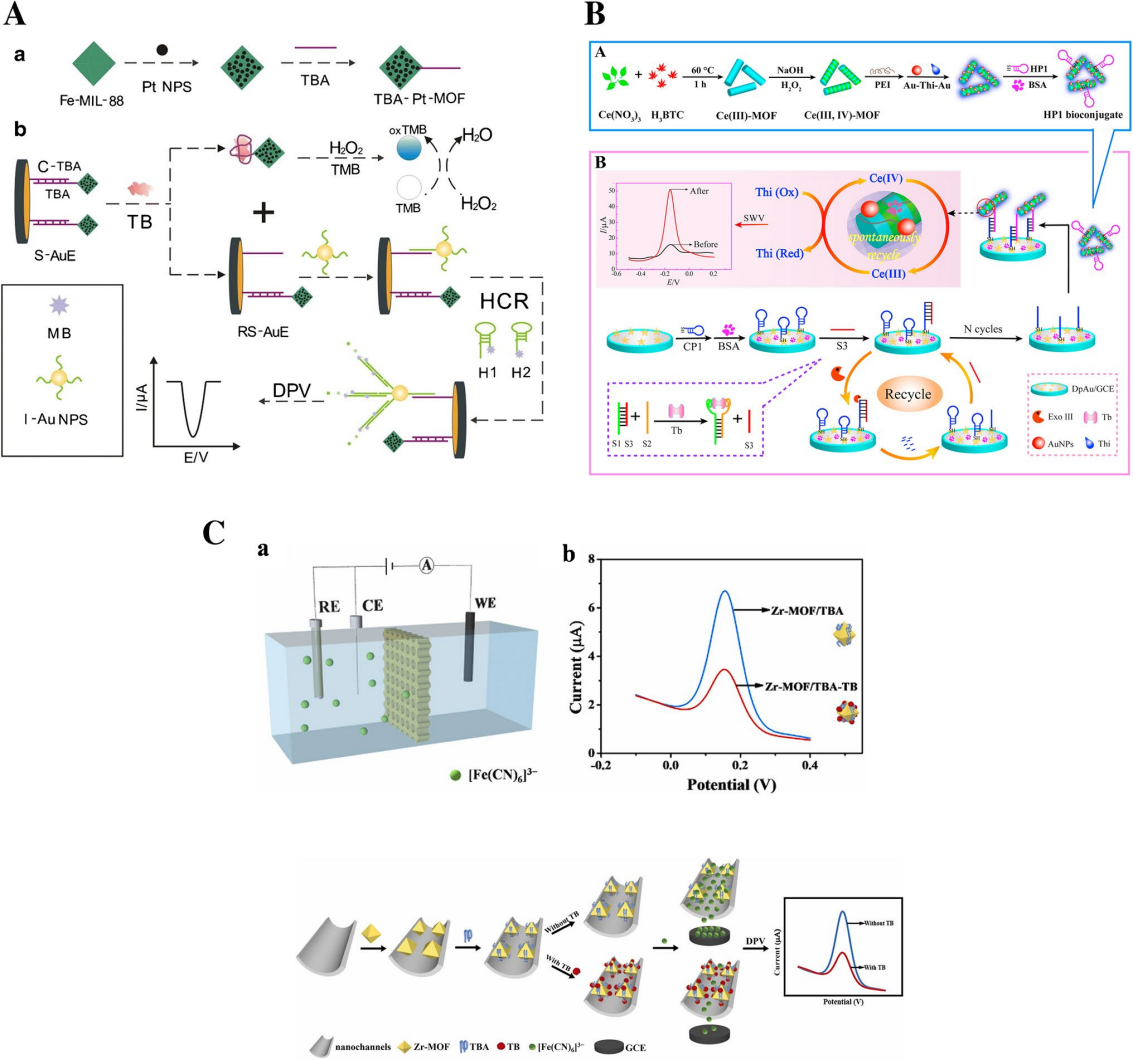
(**A**) Schematic illustration of a dual electrochemical and colorimetric sensing platform for thrombin detection based on Pt-functionalized MOF. Adapted from [89], with permission from the *Journal of Microchimica Acta*, 2019. (**B**) Schema of stepwise fabrication of dual-amplified electrochemical aptasensor for the detection of thrombin by Ce (III, IV)-MOF. Adapted from [90], with permission from *Biosensors and Bioelectronics*, 2018. (**C**) Schematic representation of an electrochemical aptasensor for thrombin detection based on Zr-MOF functionalized with DPV measurement. Adapted from [91], with permission from the *Journal of Sensors and Actuators B: Chemical*, 2023

In another study, Yang W. and coworkers [89] designed an innovative dual-readout electrochemical-colorimetric aptasensor for thrombin detection, utilizing the synergetic features of Fe-MIL-88 MOF functionalized with Pt nanoparticles (Pt-Fe-MOF). The innate electroactivity and strong peroxidase-mimicking catalytic activity were provided by the Fe^3^⁺ redox centers in MIL-88 and the embedded Pt nanoparticles, respectively. This multifunctional design improved accuracy, versatility, and reliability by enabling both colorimetric and electrochemical signal readouts within a single sensing platform. In the colorimetric channel, the thrombin-binding aptamer was covalently immobilized into the Pt-Fe-MOF (TBA-Pt-Fe-MOF), forming a biorecognition-catalyst complex. Upon the introduction of the target, Pt-Fe-MOF promoted the oxidation of chromogenic substrate 3,3′,5,5′-tetramethylbenzidine (TMB) in the presence of H₂O₂. This reaction yielded a detectable blue-colored oxidized TMB product both by UV–vis spectroscopy and to the naked eye, which allowed for the quick visual determination of thrombin on-site without the need for specialized equipment. For the electrochemical channel, a gold electrode functionalized with complementary TBA strands (C-TBA). The C-TBAs were maintained in hybridization with the aptamer on the Pt-Fe-MOF, keeping the catalytic complex immobilized in the absence of thrombin. Through the binding of thrombin, the aptamer preferentially interacted with its target, dissociating from C-TBA and releasing the TBA-Pt-Fe-MOF into solution, enabling the colorimetric catalysis of TMB/H_2_O_2_ and on-site visual detection of thrombin. Then, the dissociated C-TBAs on the electrode hybridized with gold nanoparticle-enriched initiation strands (I-AuNPs), which initiated the hybridization chain reaction (HCR) with two methylene blue-labeled hairpins (MB-H1 and MB-H2). The resultant long double-stranded DNAs carried several MB electroactive tags, significantly amplifying the differential pulse voltammetry (DPV) signal (Fig. 4(A)). The combination of this signal-off-to-on cascade in the electrochemical pathway with Pt-Fe-MOF catalysis in the colorimetric pathway enabled exceptional analytical performance. The electrochemical mode achieved a detection limit of 0.33 fM over a dynamic range of 1 fM–10 nM, while the colorimetric mode yielded an LOD of 0.17 pM across 0.5 pM–1 nM. The dual-readout platform enhanced reliability through cross-validation of results and extended the application potential from lab-based to portable and visual testing. The Pt-Fe-MOF exhibited different functions, including providing a large surface area for dense aptamer immobilization via Au–S, catalytic amplification through the peroxidase-like behavior of Pt NPs, and coordination interactions, as well as an innate redox-active signal source from Fe^3^⁺/Fe^2^⁺ cycling. Through the combination of Pt-Fe-MOF nanocatalysts, AuNP-mediated DNA assembly, and HCR-based nucleic acid amplification, this platform reached great selectivity, femtomolar sensitivity, reproducibility, and multimodal detection flexibility, aligning with the principles of next-generation aptasensor design [89].

A very sensitive electrochemical aptasensor for thrombin determination was designed by Yu H. et al. [90] using a dual signal-amplification technique in combination with a mixed-valence Ce^3^⁺/Ce^4^⁺-MOF tag. The novel aspect of this platform is the application of an electrocatalytic label, Ce(III, IV)-MOF, for thionine (Thi), in which the intrinsic Ce(III, IV) redox cycling allowed constant electron transfer without requiring additional chemical substrates. This inherent redox action, combined with the structural stability of the MOF scaffold, significantly amplified signal intensity and ensured robustness and repeatability. The detection process combined DNA strand displacement triggered by proximity binding with exonuclease III (Exo III)-assisted target recycling, providing two levels of signal amplification, primary and secondary. Specifically, upon interaction with thrombin, the two aptamers (S1 and S2) linked to the protein cause the third strand (S3) to be displaced. This shift caused capture probe 1 (CP1) to open, resulting in the formation of a double-stranded DNA substrate that Exo III could digest. Exo III preferentially cleaved these duplexes, releasing S3 to restart the cycle and generating numerous single-stranded CP1 (ss-CP1) sequences in the process. These ss-CP1 sequences were then combined with a hairpin probe (HP1) bioconjugated to Au-Thi-Au@Ce(III, IV)-MOF nanoparticles (Fig. 4(B)). The AuNPs improved conductivity and provided a stable substrate for probe attachment, while the Ce-MOF-Thi complex increased the electrochemical signal through catalytic turnover. This synergistic mechanism delivered a detection limit of 0.06 fM and a broad linear range from 0.1 fM to 10 nM. The aptasensor also demonstrated high specificity against non-target proteins, stability over repeated measurements, strong reproducibility, and effective performance in real biological samples, underscoring its potential for practical clinical application [90].

In the work of Xue and colleagues [91], a regenerative and sensitive electrochemical aptasensing platform for thrombin detection was designed based on zirconium-based MOF (Zr-MOF) functionalized solid-state nanochannels (SSNs). On the inner surface of the nanochannels, a Zr-MOF layer was formed directly through in situ growth, resulting in strong interfacial adhesion and uniform coverage that protected the structural integrity of the SSN. The abundant metal nodes, large surface area, and accessible coordination sites of Zr-MOF facilitated the effective immobilization of aptamers by generating stable Zr-O-P covalent bonds between the phosphate groups of the aptamers and Zr^4^⁺ centers, thereby positioning the aptamers precisely within the nanochannel walls for optimal target capture and signal transduction. The detection mechanism relied on monitoring the flux of the redox probe [Fe(CN)₆]^3^⁻ through the nanochannels (Fig. 4(C)). Due to the negligible nonspecific adsorption of ferricyanide to Zr-MOF surfaces, any alteration in probe signal can be associated with the interaction between aptamer and thrombin. Following the binding of the target, the thrombin-aptamer complex formed a steric barrier within the nanochannels, resulting in a significant reduction in ferricyanide transport. A measurable decrease in redox current was observed upon this reduction, facilitating quantitative, label-free detection of thrombin. A standout feature of this platform was its regenerative capability. The unsaturated Zr centers enabled the aptamer-thrombin complex to be released in a controlled way by competitive replacement with free phosphate ions. The bound aptamers and targets were removed, and the binding sites were restored to immobilize fresh aptamers while preserving the nanochannel structure. The sensor performance over 11 repeated cycles was maintained by the regeneration process with a relative standard deviation (RSD) of less than 1.8%, demonstrating the durability and cost-effectiveness of the platform. The Zr-MOF-SSN aptasensor obtained a linear range of 10 fM to 10 nM and a LOD of 4.0 fM. Its selectivity, sensitivity, label-free operation, and regeneration ability make it a robust multi-use biosensing platform for detecting thrombin and potentially other therapeutically relevant biomarkers [91].

Lastly, Jiang J. and coworkers [92] suggested a highly sensitive electrochemical aptasensor for thrombin determination using a platinum-coordinated titanium-based porphyrinic MOF (Ti-MOF-Pt). The MOF was formed of Pt(II) tetrakis(4-carboxyphenyl)porphyrin (PtTCPP) ligands, with a single Pt atom embedded in the porphyrin macrocycle through strong coordination with its four pyrrole nitrogen atoms, thereby resulting in catalytic stability and structural rigidity. The Ti-oxo clusters, acting as metal nodes, were linked with these porphyrinic ligands, providing effective charge-transfer ways throughout the framework and high structural stability. The single-atom Pt sites, as redox-active centers, resulted in the enhancement of electron-transfer kinetics, allowing for sensitive electrochemical measurements. A thrombin-binding aptamer and a capture probe were immobilized onto the Ti-MOF-Pt-modified GCE surface to create a sensing interface. The interfacial characteristics of the electrode were changed noticeably by this multi-component modification. A reduction in peak current was observed due to the immobilization of negatively charged DNA strands, leading to electrostatic repulsion against the [Fe(CN)₆]^3^⁻/^4^⁻ redox probe. This verified that the sensing layer had been properly formed. An electroactive label, methylene blue (MB), was intercalated into the double-stranded DNA created by the capture probe and its complementary aptamer sequence. The detection process was based on a conformational change in the aptamer induced by the target. The aptamer firmly bound to its target in the presence of thrombin, leading to the splitting of the double-stranded DNA and release of the embedded methylene blue into solution. As a result, the MB-related current decrease was determined using cyclic voltammetry (CV) and electrochemical impedance spectroscopy (EIS), and the change in signal was directly correlated with thrombin concentration. To optimize performance and minimize nonspecific interactions, the factors such as aptamer loading density, hybridization duration, and incubation period were investigated. Under optimal conditions, the aptasensor exhibited a linear dynamic range from 4 pM to 0.2 μM and an LOD of 1.3 pM. The platform also demonstrated high selectivity, with negligible interference from structurally unrelated proteins, including human serum albumin, bovine serum albumin, lysozyme, and immunoglobulin. Furthermore, the Ti-MOF-Pt-modified electrodes demonstrated good reproducibility across multiple fabrications and measurements, underscoring the stability and robustness of the porphyrinic MOF architecture. The Ti-MOF-Pt-based aptasensing platform offers dual advantages, including increased electron-transfer efficacy, rapid redox reactions, and stable sensor performance, thanks to the high density of catalytic sites provided by embedded Pt sites within the porphyrin rings, as well as sturdy charge-transport pathways formed by the Ti-oxo clusters. Hence, an effective sensing interface was developed based on this synergetic combination and aptamer-driven molecular recognition, proposing a robust design for next-generation MOF-based aptasensors with reliable sensitivity and selectivity in complex biological media [92].

### Bimetallic MOFs exhibiting synergistic enhancement

Bimetallic MOFs exhibiting synergistic enhancement have become central to the advancement of next-generation thrombin aptasensors.

These designs incorporate two distinct metal species rather than utilizing a single metal, which are incorporated either through hybrid architectures or within the MOF scaffold. These platforms leverage the synergistic effects and complementary attributes that outperform monometallics. In general, one of the metals supplies innate catalytic or redox activity; however, the other offers a strong affinity for biomolecules or high conductivity. Additionally, these arrangements enhance aptamer immobilization, electrochemical signal amplification, and faster electron transfer. Hence, this improves both sensitivity and selectivity, and also expands the design flexibility of MOF-based biosensors [53, 86, 93]

**Fig. 5:**
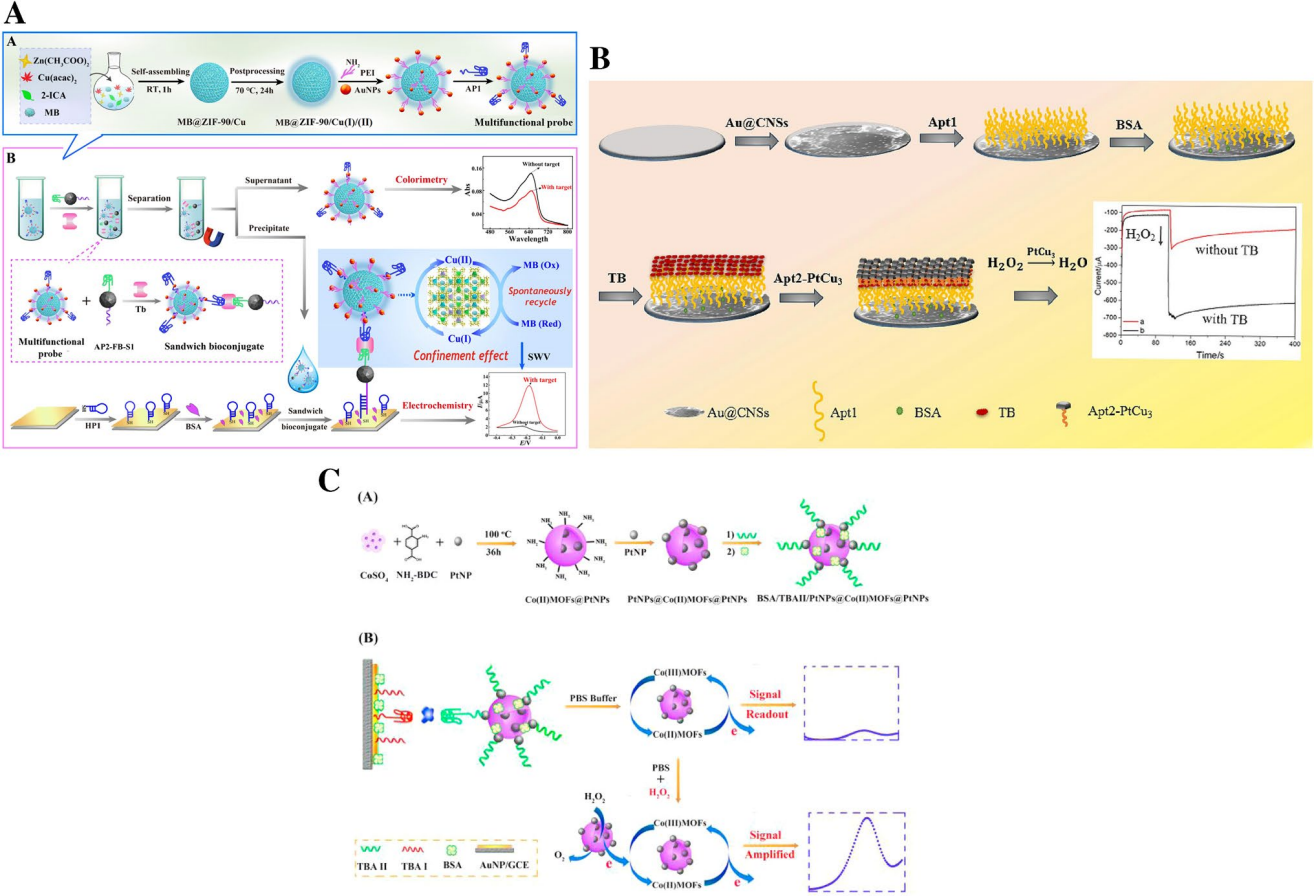
(**A**) Illustrating schema of electrochemical-colorimetric dual-signal aptasensing platform; (a) steps involved in the preparation of multifunctional probe MB@ZIF-90/Cu(I)/(II), and (b) construction process of bimodal aptasensor and signal enhancement strategy for thrombin detection. Adapted from [94], with permission from the *Microchemical Journal*, 2024. (**B**) Schematic representation of the sandwich-type electrochemical aptasensor fabrication process. Adapted from [95], with permission from the *Journal of Electroanalytical Chemistry*, 2024. (**C**) Illustration of the electrochemical thrombin aptasensor design, including (a) the preparation steps of the TBA II bioconjugates and (b) the signal amplification mechanism, allowing thrombin detection. Adapted from [96], with permission from the *Journal of Talanta*, 2017

A representative example from late 2024 is the development of a dual-mode colorimetric–electrochemical aptasensing platform for sensitive thrombin determination through a combination of an effective inherent signal amplification process within a sandwich-type architecture [94]. The core of this design was a multifunctional probe, MB@ZIF-90/Cu(I, II), in which the redox dye methylene blue was encapsulated within the pores of a mixed-valence Cu(I)/Cu(II) zeolitic imidazolate framework (ZIF-90). MB served a dual role as both electroactive and chromogenic reporter, while the Cu(I)/Cu(II) nodes catalyzed its redox cycling spontaneously, even in the absence of added substrates. The built-in catalytic activity of this design was further increased by the confinement effect within the MOF’s microporous structure, which intensified Cu–MB interactions and increased electron-transfer efficiency. Electrodeposition of AuNPs onto MB@ZIF-90/Cu(I, II) enabled aptamer-driven molecular recognition. It provided highly conductive sites for the covalent immobilization of TBA1 (AP1) through Au–S bonds, resulting in AP1/Au/MB@ZIF-90/Cu(I, II). Simultaneously, AP2-FB-S1 was created by co-functionalization of TBA2 (AP2) with a single-stranded capture DNA (S1) onto ferric oxide magnetic beads (FB). Upon thrombin presentation, a sandwich-type complex was generated via thrombin-mediated molecular bridging, which assembled AP1/Au/MB@ZIF-90/Cu(I, II) with AP2-FB-S1. After magnetic separation, the complexes were immobilized on a hairpin-probe-modified GCE (AuNPs/HP1/GCE) for electrochemical readout (Fig. 5(A)). Concurrently, the supernatant containing unbound MB@ZIF-90/Cu(I, II) probes was used for colorimetric analysis, exploiting the visible absorbance change of MB upon redox cycling. As a result, the electrochemical–colorimetric dual-readout aptasensor provided complementary quantitative outputs, with linear ranges of 1 pM–10 μM and 0.1 nM–100 μM and LODs of 0.3 pM and 0.03 nM for electrochemical and colorimetric detection, respectively. The dual-mode platform was demonstrated to offer several benefits, including the reduction of false positives through internal cross-validation, retention of high reproducibility and stability, and applicability to real human serum specimens. The ZIF-90 MOF played a dual role as both a catalytic reactor and a nanocarrier, co-localizing MB reporters and Cu redox centers within its pores. Meanwhile, the aptamers provided the required selectivity for thrombin, combining molecular recognition and catalytic amplification in a single nanoscale assembly [94].

In the same year, Xiong W. et al. [95] introduced a bimetallic MOF-derived sandwich-type electrochemical aptasensor for sensitive thrombin detection, leveraging a dual signal amplification strategy based on PtCu₃ alloy nanoparticles and Au-decorated carbon nanosheets (Au@CNSs) (Fig. 5(B)). The platform was created from a thermally carbonized 2D-porphyrinic MOF, Cu-TCPP (Cu-tetrakis(4-carboxyphenyl)porphyrin), to produce conductive 2D-CNSs (carbon nanosheets) while maintaining the structural order essential for a large surface area and porosity. Afterwards, Au@CNSs were produced by decorating CNSs with gold nanoparticles, which increased conductivity and provided several Au–S binding sites for aptamer immobilization. In the meantime, PtCu₃ alloy nanocrystals were synthesized and leveraged the electrocatalytic activity of Cu and Pt toward H₂O₂ reduction, a commonly employed reaction for electrochemical signal production. The dual-metal alloy facilitated fast electron-transfer kinetics and supplied high catalytic efficacy. The aptasensor was based on a sandwich format, in which a thiolated TBA, serving as a capture probe (Apt1), was covalently immobilized to the Au@CNS-modified GCE via an Au–S linkage. Apt2-PtCu₃ was prepared by integrating the detection probe (Apt2) with PtCu₃ alloy nanoparticles, which led to amplification of the catalytic signal. The target protein selectively interacted with both Apt1 and Apt2 in the presence of thrombin, resulting in linkage of the PtCu₃-Apt2 probe with the Au@CNSs-modified electrode surface to generate a stable sandwich-type biosensing interface. The near-electrode positioning of the PtCu₃ catalytic sites resulted in a significant increase in H₂O₂ electroreduction, reduction current amplification, and sensitive thrombin determination. Performance evaluation revealed a broad linear detection range from 0.01 pM to 32 nM and a detection limit of 0.005 pM, attributed to the synergistic effects of the high surface area Au@CNSs and the superior catalytic activity of PtCu₃ nanoparticles. Selectivity and recovery experiments in diluted human serum demonstrated negligible cross-reactivity with non-target proteins, indicating potential use in clinical diagnostics. Despite good analytical performance, multiplexed synthesis and functionalization processes may restrict large-scale production [95].

Yang and colleagues [96] developed a sensitive electrochemical aptasensor for thrombin detection within the scope of bimetallic MOFs designed to exploit synergistic enhancement by synthesizing platinum nanoparticle-functionalized cobalt-based MOF (PtNPs@Co(II)MOFs@PtNPs). A composite solvothermal procedure was employed to synthesize the nanocomposite. PtNPs were encapsulated inside amino-functionalized Co(II)MOFs with Co^2^⁺ as the central ion and 2-amino-terephthalic acid (NH₂-H₂BDC) as the ligand. This was followed by post-synthetic deposition of an additional PtNP layer on the outer surface of the MOF through Pt–N coordination, resulting in the final PtNPs@Co(II)MOFs@PtNPs structure. This dual incorporation strategy provided the hybrid with a hierarchical architecture, in which the inner PtNPs facilitated internal electron transport. At the same time, surface-bound PtNPs maximized catalytic site availability and increased surface area, enabling the dense immobilization of aptamer TBA II and forming aptamer-nanocomposite bioconjugates. For electrode construction, the GCE was electrochemically decorated with AuNPs and subsequently incubated with TBA I. After thrombin introduction, the resulting TBA II-PtNPs@Co(II)MOFs@PtNPs bioconjugates were assembled onto the electrode in a sandwich configuration with the target thrombin, where thrombin recognition produced an electrochemical output stemming directly from Co(II)/Co(III) redox cycling (Fig. 5(C)). The application of cyclic voltammetry to evaluate the progressive modification of the electrode with the [Fe(CN)₆]^3^⁻/^4^⁻ redox probe showed successful surface functionalization. The PtNPs@Co(II)MOFs@PtNPs acted as aptamer carriers and also as inherent redox tags, enabling charge creation and electron transfer through the Co(II)/Co(III) redox couple. Moreover, a synergistic system was generated by combining Co-MOFs with PtNPs, in which PtNPs served as strong electrocatalysts for H₂O₂ oxidation, thereby accelerating electron transfer between the electrode and Co centers, and significantly amplifying the electrochemical signal in the presence of H₂O₂ without the need for external mediators. PtNPs@Co(II)MOFs@PtNPs enhanced signal amplification remarkably in comparison to Co(II)MOFs loaded with a single Pt component, highlighting the significance of bimetallic synergy. The aptasensor demonstrated LOD of 0.33 fM and a linear range from 0.1 pM to 50 nM. The designed aptasensor also showed selectivity against interfering proteins (PCT, Cys, Hb, influenza antigen), over 99% signal preservation after 20 days at 4 °C, and stability and reproducibility (RSD < 5.1%). Moreover, spiked serum analyses with RSDs of 2.1–3.2% verify its practical application in complex biological samples. In this study, the simultaneous enhancement of electron transfer, catalytic turnover, and aptamer loading was achieved through synergistic cooperation between Pt catalytic centers and Co redox-active nodes in a bimetallic MOF, thereby facilitating selectivity, sensitivity, and stability in thrombin detection. This approach utilizes bimetallic MOFs with redox-active centers to develop next-generation aptasensors for clinical diagnostics and broader bioanalytical applications [96].

A summary of the MOF-based thrombin aptasensors in Table 1 illustrates how changes in the composition of the metal node, hybrid functionalization, and amplification processes directly affect the analytical outcome. 509-MOF and Fe-MIL-88 (hemin@MOFs), as monometallic MOFs with intrinsic redox activity, leverage the coordination and catalytic capabilities of their Zr(IV) and Fe(III) nodes to boost aptamer immobilization and electron transfer. The Zr-based 509-MOF used strong Zr-O-P covalent bonding to allow stable DNA immobilization with a LOD of 0.37 pg/mL (0.01 pM), while the Fe-MIL-88 hybridized with glucose oxidase and AuNPs and reached sensitivity with a LOD of 0.068 pM. However, in bimetallic MOF composites, the integration of numerous catalytic and structural components results in exceptional synergistic improvement [97, 98]. For instance, MoS₂-based hybrids and 3D-DNA nanostructures coupled with AuNPs@Fe-MIL-88 acquired a LOD of 59.6 fM by using both the catalytic conductivity of MoS₂ and the high DNA loading capacity of Fe-MOFs. Likewise, the integration of PtPd nanoparticles, AuNPs, and hairpin DNA with Co-MOF via a nicking enzyme signaling amplification (NESA) mechanism simultaneously amplified catalysis and recognition, attaining a LOD of 0.32 pM [99, 100].

**Table 1:** Representative monometallic and bimetallic MOF-based electrochemical aptasensors for thrombin detection

Type of MOF	Metal node	Hybrid/functional components	Amplification mechanism	Classification	Electrode type	Aptasensor format	Linear range	LOD	Ref
Fe-MIL-88 (hemin@MOFs)	Fe(III)-oxo clusters with hemin	AuNPs + glucose oxidase	Enzyme catalysis + Au–N bonding/self-assembly for electron-transfer	Monometallic MOFs with intrinsic redox activity	GCE	Sandwich-type	0.0001–30 nM	0.068 pM	[98]
509-MOF	Zr(IV) clusters	Zr(IV) ion crosslinking	Zr-O-P covalent coordination between DNA phosphate groups and Zr nodes, enhancing charge transfer	Monometallic MOFs with intrinsic redox activity	Au electrode	–	0.001–0.5 ng/mL	0.37 pg/mL (≈0.01 pM)	[97]
Co-MOF	Co(II) clusters	PtPd NPs + hairpin DNA (HP3) + AuNPs	Nicking enzyme signaling amplification (NESA) coupled with PtPd catalysis for synergistic signal gain	Bimetallic MOFs exhibiting synergistic enhancement	GCE	–	1 pM – 30 nM	0.32 pM	[100]
AuNPs@Fe-MIL-88	Fe(III)-oxo clusters	3D DNA nanostructure (NTH) + AuNPs@IL-MoS₂	DNA nanostructure-assisted target capture with MoS₂-based electrocatalysis, enhancing electron-transfer	Bimetallic MOFs exhibiting synergistic enhancement	GCE	Sandwich-type	0.298–29.8 pM	59.6 fM	[99]

### MOF-derived nanocomposites leveraging post-synthetic modifications

MOF-derived nanocomposites leveraging post-synthetic modifications depict a promising approach to address the inherent boundaries, such as restricted catalytic activity and low conductivity of pristine MOFs.

MOFs can be transformed or merged into hybrid materials with improved electrochemical performances through post-synthetic modification and thermal transformation. The calcination of MOFs, or their combination with catalytic and conductive nanomaterials, results in compounds with increased active-site density, high conductivity, and structural stability. These designed nanocomposites supply robust, efficient platforms for signal enhancement in thrombin aptasensors, as described by the following examples [66, 73, 74] 

**Fig. 6:**
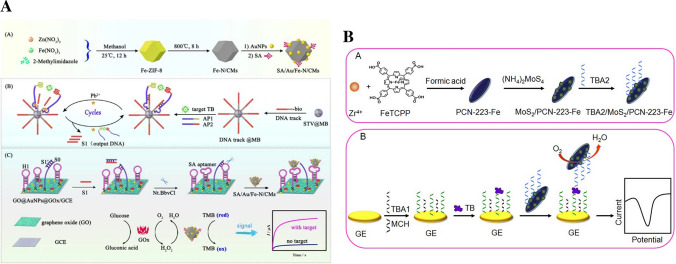
(**A**) Diagrammatic demonstration of the biosensor preparation steps, including (a) synthesis of a functionalized SA/Au/Fe-N/CMs enzyme; (b) application of DNAzyme for amplification of target cycling for abundant output DNA; trigger of effective enzyme cascade electrocatalysis by output DNA for target determination and mechanism of response. Adapted from [101], with permission from the *Journal of Microchimica Acta*, 2023. (**B**) Illustration of fabrication steps and mechanism of sandwich-type electrochemical aptasensor for thrombin detection using TBA2/MoS_2_/PCN-223-Fe. Adapted from [102], with permission from the *Journal of The Electrochemical Society*, 2020

For instance, in 2024, Xie P. et al. [101] synthesized Fe–N-doped carbon nanomaterials (Fe–N/CMs) as high-performance nanozymes, derived from controlled carbonization of Fe-ZIF-8 MOF precursors. The synthesized Fe–N/CMs contained atomically dispersed Fe–N active sites embedded in a conductive carbon matrix, providing fast electron transfer, strong peroxidase-like catalytic behavior, and sturdy structural stability. To enhance catalytic and biorecognition capabilities, the functionalization of Fe–N/CMs with AuNPs and their conjugation with streptavidin (SA) yielded SA/Au/Fe–N/CMs, which showed increased surface reactivity and conductivity. The thrombin biosensing platform employed an enzyme-cascade catalytic amplification system that integrated glucose oxidase (GOx) with SA/Au/Fe–N/CMs and a target-induced DNA walker. The first step in the DNA walker system was the creation of track DNA@MB by immobilizing biotin-labeled track DNA onto streptavidin-coated magnetic beads (MB). Upon addition of various thrombin concentrations, two thrombin-binding aptamers (AP1 and AP2), and Pb^2^⁺ (as a DNAzyme cofactor), thrombin binding caused an output DNA strand (S1) to be released by DNAzyme-mediated strand displacement. To prepare the electrode, a GO@AuNPs@GOx composite was used to modify a GCE, which combined the enzymatic activity of GOx with the high conductivity of AuNP-decorated graphene oxide. Thereafter, the surface was hybridized with a hairpin probe (H1) and S0/S1′ duplex comprising inactivated arm DNA (S0) and blocker DNA (S1′). The introduction of nicking endonuclease Nt.BbvCI and output DNA S1 onto the modified GCE surface resulted in S1 and S1′ hybridization, which released the S0 “swing arm” strand. This arm DNA induced a DNA walker process, cyclically cleaving H1 on the electrode surface to expose binding sites to form the SA aptamer. Finally, the SA/Au/Fe–N/CMs nanozyme complex was integrated with SA aptamer sites via strong SA-biotin interactions, forming the complete sensing interface (Fig. 6(A)). Hydrogen peroxide was created by the oxidation of glucose utilizing GOx. Then, it was degraded by the Fe–N/CM nanozyme, resulting in an enhanced electrochemical signal. This dual-enzyme cascade accomplished thrombin detection with a limit of detection of 3 fM and a broad linear range from 0.01 pM to 1 nM. The platform showed promising potential for clinical diagnostic applications, with good selectivity, stability, and reproducibility [101].

Zhang J. and colleagues [102] reported an ultrasensitive sandwich-type electrochemical aptasensor using a MoS₂ nanoparticle-loaded PCN-223-Fe composite as a signal amplifier for the detection of thrombin. PCN-223-Fe is a porphyrinic MOF containing Zr^4^⁺ ions and iron-porphyrin linkers that act as robust metal nodes and intrinsic redox centers for providing effective charge-transfer routes and stability. To increase the surface area for further functionalization, the material was prepared in a spindle-like morphology. The hybrid MoS₂/PCN-223-Fe composite was formed by loading MoS₂ nanoparticles onto the PCN-223-Fe surface to improve catalytic activity and conductivity. To fabricate the aptasensor, the gold electrode surface was modified with the capture probe TBA1 via its terminal thiol group, forming a covalent Au–S bond. TBA2/MoS₂/PCN-223-Fe was formed by conjugating TBA2, the signal probe, to the MoS₂/PCN-223-Fe composite. The target protein was selectively bound to TBA1 and TBA2 in the presence of thrombin, creating a stable sandwich complex between the MoS₂/PCN-223-Fe probe and the electrode surface. The highly electrocatalytic MoS₂/PCN-223-Fe was located in this close arrangement to the electrode interface, where it effectively catalyzed oxygen reduction (Fig. 6(B)). Based on electrochemical analyses, increasing thrombin concentration led to a proportional increase in the current of the catalytic oxygen reduction reaction, providing a quantitative readout. With a detection limit of 0.03 pM and a broad range of 0.1 pM to 100 nM, the aptasensor reflected the synergistic contributions of MoS₂-mediated electron-transfer and porphyrinic Fe redox sites. Additionally, it showed good specificity, reproducibility, and stability across multiple measurements. The fabricated platform demonstrates how nanomaterial-MOF hybrids can efficiently combine nanoscale catalytic amplifiers with the inherent redox chemistry of MOFs to achieve highly sensitive biosensing performance [102].

**Fig. 7:**
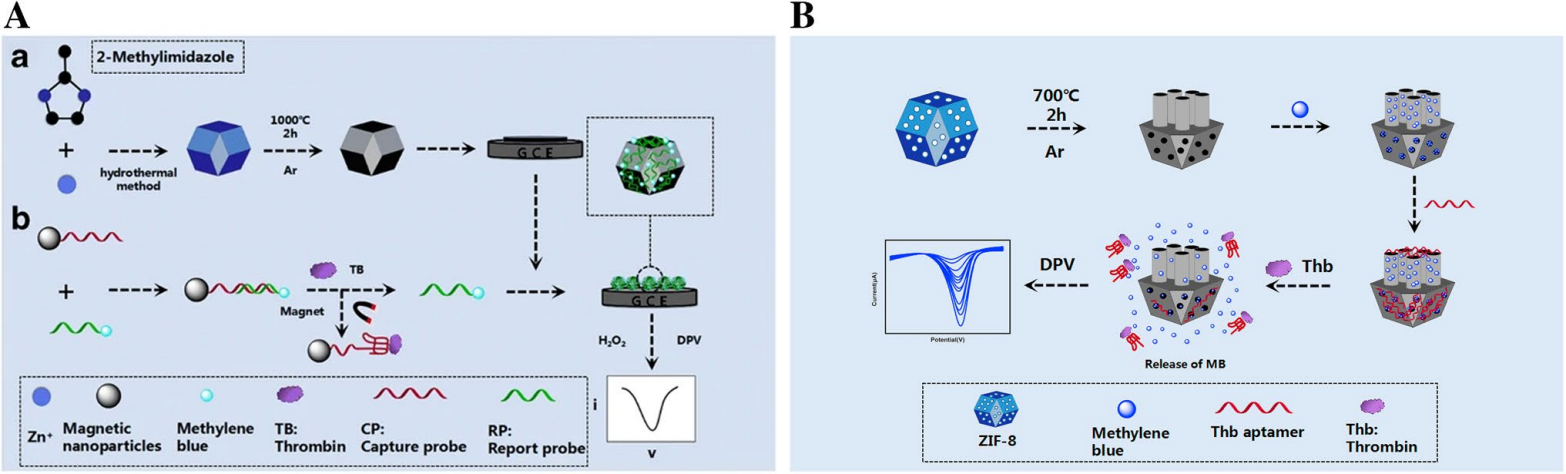
(**A**) A schema of (a) synthesis of Z-1000 porous carbon nanomaterial and (b) the Z-1000-based electrochemical sensing platform for determination of thrombin. Adapted from [103], with permission from the *Journal of Microchimica Acta*, 2019. (**B**) A schematic illustration of a homogeneous electrochemical aptasensor for thrombin detection using a Thb-specific aptamer serving as a bio-gate to control the signal molecules release from a target-responsive porous carbon nanocontainer derived from ZIF-8. Adapted from [104] with permission from the *Journal of Biosensors and Bioelectronics*, 2020

In another study, Liu Q. and coworkers [103] fabricated a thrombin-specific electrochemical aptasensor in 2019 that used Z-1000, a carbonized derivative of ZIF-8 (zinc(II)−2-methylimidazole MOF), in combination with magnetic nanoparticles (MNPs) for signal amplification. Z-1000 was a porous carbon nanomaterial characterized by improved conductivity and a high surface area, making it suitable for electrode modification. Its high surface area enabled effective nucleic acid adsorption through π–π stacking between nucleobases and aromatic carbon planes. For the fabrication of the aptasensor, CP/MNP complexes were created by covalent conjugation of carboxyl-functionalized MNPs to capture probes (CPs) containing 5′-amino-modified thrombin-specific aptamer sequences via amide bonds. In the initial sensing arrangement, methylene blue-labeled electroactive DNA strands, serving as reporter probes (RPs), were hybridized with CPs to form RP/CP/MNP complexes. Upon the presence of thrombin, the RP strand was competitively displaced from the RP/CP/MNP complex by the selective binding of the CP aptamer and thrombin. Strong π–π stacking interactions enabled selective adsorption of free MB-tagged RPs onto the Z-1000-modified GCE, thereby facilitating effective accumulation of the electroactive species at the electrode surface. As the redox-active reporter, MB produced a measurable current proportional to thrombin concentration (Fig. 7(A)). This indirect signal amplification enabled sensitive detection with a LOD of 0.8 fM and a wide dynamic range between 10 fM and 100 nM. The developed aptasensing platform demonstrated high reproducibility, good selectivity, and stability. The combination of magnetic separation for target isolation and MOF-derived carbon nanostructures for signal amplification underscores a promising approach for sensitive aptamer-based biosensing [103].

Finally, research by Ren and colleagues [104] led to the development of a homogeneous electrochemical aptasensor for thrombin detection using an aptamer-gated ZIF-8 (zeolitic imidazolate framework-8)-derived porous carbon nanocontainer, termed Z-700. The operation of this system relies on the target-triggered release of an electroactive reporter mechanism. After carbonization of ZIF-8 at 700 °C, the nanocontainer was produced in which the crystalline MOF converted into a conductive, graphene-like porous carbon material with a high surface area, large pore volume, a π-electron-rich structure, and biocompatibility. These attributes allowed the efficient encapsulation of the electroactive dye methylene blue within the pores and the adsorption of TBA via π-π stacking with nucleobases. In the absence of the target, the aptamer played a role as a molecular bio-gate, retaining the MB within Z-700 and preventing its release. Upon thrombin introduction, specific binding between thrombin and the aptamer caused formation of a thrombin-aptamer complex that underwent conformational rearrangement, leading to aptamer displacement from the Z-700 surface. The conformational changes induced pore gate opening, thereby triggering the release of MB into solution (Fig. 7(B)). The released MB was measured by DPV at a screen-printed electrode, showing a significant increase in the reduction current proportional to the thrombin concentration. Dependence only on analyte-induced release in solution streamlined procedures and enhanced reproducibility in the homogeneous assay format due to the elimination of labeling and electrode surface modification steps. In the presence of thrombin, the developed platform exhibited 8.6-fold signal amplification with a LOD of 0.57 fM and a dynamic range of 1 fM to 1 nM. This performance stemmed from robust but reversible aptamer adsorption on Z-700, its high reporter-loading capacity, and the effective electrochemical response of methylene blue. The designed platform exhibited great selectivity toward BSA, HSA, and lysozyme, while stability studies showed slight signal reductions of < 6.70% for the blank and < 3.71% for the target after extended storage at 4 °C. Moreover, reproducibility investigations revealed low relative standard deviations of 7.81% inter-sensor and 2.30% intra-sensor. The aptasensor attained recoveries of 95.65–98.32% with RSDs < 7.45% in spiked human serum experiments, verifying its reliable performance in complex biological media. The study presented that the application of ZIF-8-derived porous carbon as a nanocontainer, in combination with aptamer bio-gating, offers a sensitive, reliable, and rapid system for biomolecular determination, with great promise for clinical diagnostics [104].

In conclusion, recent advancements in the fabrication of electrochemical aptasensors based on metal–organic frameworks for thrombin detection have highlighted the efficacy of tailored framework design in electrochemical signal generation and amplification. These strategies demonstrate the importance of regulation of metal composition, conductivity, and amplification mechanisms, which define sensor performance and practical applicability. Even though the combination of aptamer selectivity and the structural tunability of MOFs has enabled the development of powerful thrombin biosensing platforms, ongoing research is necessary to enhance reproducibility and robustness, as well as to facilitate scalable construction for clinical implementation [35, 60].

## Translational pathways for MOF-aptamer biosensors: from lab to commercialization-challenges and opportunities

Although hybrid MOF-aptamer electrochemical biosensors perform well analytically in laboratory settings, translating them into clinical and commercial diagnostics demands overcoming obstacles such as stability, cost, scalable fabrication, regulatory approval, and real-world integration. There are some important challenges, including short-term stability, the expense of noble metals, and the need for repeatable large-scale aptamer and MOF synthesis. These restrictions are being addressed by new techniques such as low-temperature and green synthesis, replacement with abundant metals, high-throughput printing, and lyophilization or encapsulation strategies [105, 106]. Also of paramount importance is the combination of MOF-aptamer platforms with user-friendly POC technologies, such as microfluidics, screen-printed or flexible electrodes, and smartphone-readout devices, along with wash-free, facilitated assay workflows. For successful clinical translation, thorough clinical validation in complex biological matrices and early alignment with the regulatory framework are critical. Given the established applications of MOF-aptamer sensing platforms in cardiovascular and coagulation monitoring and the growing industrial interest, a multidisciplinary approach incorporating biointerface engineering, systems integration, regulatory techniques, and materials science is essential. This strategy offers a clear, practical pathway for translating MOF-based thrombin aptasensors into scalable, clinically and commercially viable diagnostic platforms [106–108].

## Conclusion and outlook

Thrombin serves vital roles not only in hemostasis and wound healing but also in inflammation, tumor progression, and systemic coagulation disorders. These wide-ranging functions highlight the significance of rapid, precise thrombin detection for early diagnosis and efficient clinical treatment. Over the past few years, the advancement of biosensors, especially electrochemical aptasensors, has accelerated remarkably, driven by the demand for highly sensitive, portable, and real-time diagnostic tools.

This review underscores the synergy between metal–organic frameworks and thrombin-binding aptamers, enabling the development of highly sensitive and selective electrochemical aptasensors. The characteristics of MOFs, including redox-active metal centers, rich coordination chemistry, and tunable porosity, enable effective aptamer immobilization, signal amplification, and stable electrode interfaces. The unique merits of MOF-based aptasensors over traditional platforms are highlighted by mechanistic insights into amplification strategies, metal–ligand coordination, and metal core effects. Current developments include MOF-derived nanocomposites with customized functions, bimetallic MOFs with synergistic enhancement, and monometallic frameworks with inherent electroactivity, reaching femtomolar detection limits and adaptability across sandwich-type, label-free, and dual-readout systems. Overall, these advances position MOF-aptamer hybrid sensors as strong candidates for point-of-care diagnostics. Importantly, this review provides the first focused overview of thrombin-targeted MOF-aptamer platforms, addressing a critical gap in the literature and summarizing a decade of progress (2015–2025).

Looking toward the future, it will be significant for successful clinical translation to have more stable aptamers, reproducible and scalable fabrication, and comprehensive validation in complicated biological matrices. Continuous progress in aptamer engineering, metal–organic framework design, and signal amplification via multidisciplinary strategies will support the integration of these platforms into miniaturized, multiplexed, and user-friendly diagnostic devices, offering a practical roadmap for clinical and commercial translation into viable diagnostics. Addressing these challenges will enable MOF-aptasensors to transform thrombin detection and expand their utility across diverse diagnostic applications.

## Data Availability

This review did not generate any new data. All data discussed are available in the cited literature. This work comprises figures that do not belong to the authors and were reprinted with permission from the relevant references.

## References

[1] Di Cera E. Thrombin. Mol Aspects Med. 2008;29(4):203–54. 18329094 10.1016/j.mam.2008.01.001 PMC2491495

[2] Hernandez-Rodriguez N, Correa E, Contreras-Paredes A. Thrombin: a new useful factor in the early diagnosis of pulmonary metastasis. Rev Inst Nal Cancerol. 1997;43:65–75.

[3] Mir M, Vreeke M, Katakis I. Different strategies to develop an electrochemical thrombin aptasensor. Electrochem Commun. 2006;8(3):505–11.

[4] Eivazzadeh-Keihan R, Saadatidizaji Z, Maleki A, Guardia M, Mahdavi M, Barzegar S, Ahadian S. Recent progresses in development of biosensors for thrombin detection. Biosensors. 2022;12(9):767. 36140153 10.3390/bios12090767 PMC9496736

[5] Kotlarek D, Vorobii M, Ogieglo W, Knoll W, Rodriguez-Emmenegger C, Dostalek J. Compact grating-coupled biosensor for the analysis of thrombin. ACS Sens. 2019;4(8):2109–16. 31364363 10.1021/acssensors.9b00827

[6] Riccardi C, Napolitano E, Platella C, Musumeci D, Montesarchio D. G-quadruplex-based aptamers targeting human thrombin: discovery, chemical modifications and antithrombotic effects. Pharmacol Ther. 2021;217:107649. 32777331 10.1016/j.pharmthera.2020.107649

[7] Shen G, Zhang H, Yang C, Yang Q, Tang Y. Thrombin ultrasensitive detection based on chiral supramolecular assembly signal-amplified strategy induced by thrombin-binding aptamer. Anal Chem. 2017;89(1):548–51. 27958723 10.1021/acs.analchem.6b04247

[8] Fredenburgh JC, Weitz JI. Exosite crosstalk in thrombin. J Thromb Haemost. 2025. 10.1016/j.jtha.2025.01.003. 39842513 10.1016/j.jtha.2025.01.003

[9] Sun H, Wang N, Zhang L, Meng H, Li Z. Aptamer-based sensors for thrombin detection application. Chemosensors. 2022;10(7): 255.

[10] KNOWN I (1801) Diagnosis and management of nephrotic syndrome. Practitioner 2017;(261):11–15. 29020719

[11] Kim H, An Z, Jang C-H. Label-free optical detection of thrombin using a liquid crystal-based aptasensor. Microchem J. 2018;141:71–9.

[12] Yousef H, Liu Y, Zheng L. Nanomaterial-based label-free electrochemical aptasensors for the detection of thrombin. Biosensors. 2022;12(4):253. 35448312 10.3390/bios12040253 PMC9025199

[13] Lim Y, Kouzani A, Duan W. Aptasensors: a review. J Biomed Nanotechnol. 2010;6(2):93–105. 20738063 10.1166/jbn.2010.1103

[14] Mathew M, Rout CS. Electrochemical biosensors based on Ti3C2Tx MXene: future perspectives for on-site analysis. Curr Opin Electrochem. 2021;30: 100782.

[15] Maoqiang W, Xianhua S, Linxi C, Duanping S. Metal-organic framework-based electrochemical aptasensors for detecting cancer biomarkers. Journal of Holistic Integrative Pharmacy. 2022;3(2):190–205.

[16] Chandio I, Rahujo S, Chandio ZA, Rashid MA, Rehman MU, Shar ZH, Thebo KH. Recent development in metal-organic frameworks-based electrochemical aptasensors for detection of cancer biomarkers. Bioelectrochemistry. 2025. 10.1016/j.bioelechem.2025.109006. 40393088 10.1016/j.bioelechem.2025.109006

[17] Espiritu CAL, Justo CAC, Rubio MJ, Svobodova M, Bashammakh AS, Alyoubi AO, Rivera WL, Rollon AP, O’Sullivan CK. Aptamer selection against a *Trichomonas vaginalis* adhesion protein for diagnostic applications. ACS Infect Dis. 2018;4(9):1306–15. 29972299 10.1021/acsinfecdis.8b00065

[18] Jayasena SD. Aptamers: an emerging class of molecules that rival antibodies in diagnostics. Clin Chem. 1999;45(9):1628–50. 10471678

[19] Hassanpour S, Mokhtarzadeh A, Hasanzadeh M, Hejazi M, Baradaran B. Nanomaterials for use in apta-assays: analytical approach. In: de la Guardia M, Esteve-Turrillas FA, editors. Handbook of Smart Materials in Analytical Chemistry. John Wiley & Sons; 2019. pp 243–271.

[20] Khoshbin Z, Davoodian N, Taghdisi SM, Abnous K. Metal organic frameworks as advanced functional materials for aptasensor design. Spectrochim Acta A Mol Biomol Spectrosc. 2022;276:121251. 35429856 10.1016/j.saa.2022.121251

[21] Noonan PS, Roberts RH, Schwartz DK. Liquid crystal reorientation induced by aptamer conformational changes. J Am Chem Soc. 2013;135(13):5183–9. 23510322 10.1021/ja400619k

[22] Stoltenburg R, Reinemann C, Strehlitz B. SELEX—a (r) evolutionary method to generate high-affinity nucleic acid ligands. Biomol Eng. 2007;24(4):381–403. 17627883 10.1016/j.bioeng.2007.06.001

[23] Hasanzadeh M, Shadjou N, de la Guardia M. Aptamer-based assay of biomolecules: recent advances in electro-analytical approach. TrAC Trends Anal Chem. 2017;89:119–32.

[24] Karimzadeh Z, Mahmoudpour M, de la Guardia M, Dolatabadi JEN, Jouyban A. Aptamer-functionalized metal organic frameworks as an emerging nanoprobe in the food safety field: promising development opportunities and translational challenges. TrAC Trends Anal Chem. 2022;152:116622.

[25] Bock LC, Griffin LC, Latham JA, Vermaas EH, Toole JJ. Selection of single-stranded DNA molecules that bind and inhibit human thrombin. Nature. 1992;355(6360):564–6. 1741036 10.1038/355564a0

[26] Bai Y, Feng F, Zhao L, Wang C, Wang H, Tian M, Qin J, Duan Y, He X. Aptamer/thrombin/aptamer-AuNPs sandwich enhanced surface plasmon resonance sensor for the detection of subnanomolar thrombin. Biosens Bioelectron. 2013;47:265–70. 23584389 10.1016/j.bios.2013.02.004

[27] Deng B, Lin Y, Wang C, Li F, Wang Z, Zhang H, Li X-F, Le XC. Aptamer binding assays for proteins: the thrombin example—a review. Anal Chim Acta. 2014;837:1–15. 25000852 10.1016/j.aca.2014.04.055

[28] Tasset DM, Kubik MF, Steiner W. Oligonucleotide inhibitors of human thrombin that bind distinct epitopes. J Mol Biol. 1997;272(5):688–98. 9368651 10.1006/jmbi.1997.1275

[29] Troisi R, Riccardi C, de Carvasal KP, Smietana M, Morvan F, Del Vecchio P, Montesarchio D, Sica F. A terminal functionalization strategy reveals unusual binding abilities of anti-thrombin anticoagulant aptamers. Mol Ther Nucleic Acids. 2022;30:585–94. 36457701 10.1016/j.omtn.2022.11.007 PMC9707062

[30] Kong D-M, Xu J, Shen H-X. Positive effects of ATP on G-quadruplex-hemin DNAzyme-mediated reactions. Anal Chem. 2010;82(14):6148–53. 20552961 10.1021/ac100940v

[31] Samani SS, Sameiyan E, Yazdi FT, Mortazavi SA, Alibolandi M, Ramezani M, Taghdisi SM, Abnous K. Sandwich-type aptamer-based biosensors for thrombin detection. Anal Methods. 2024;16(14):1985–2001. 38502201 10.1039/d3ay02196c

[32] Hassanpour S, Niaei N, Petr J. Metal–organic frameworks-based analytical devices for chiral sensing and separations: a review (2012–2022). Chemosensors. 2022;11(1):29.

[33] Kempahanumakkagari S, Vellingiri K, Deep A, Kwon EE, Bolan N, Kim K-H. Metal–organic framework composites as electrocatalysts for electrochemical sensing applications. Coord Chem Rev. 2018;357:105–29.

[34] Yaghi OM, Li G, Li H. Selective binding and removal of guests in a microporous metal–organic framework. Nature. 1995;378(6558):703–6.

[35] Lv M, Zhou W, Tavakoli H, Bautista C, Xia J, Wang Z, Li X. Aptamer-functionalized metal-organic frameworks (MOFs) for biosensing. Biosens Bioelectron. 2021;176:112947. 33412430 10.1016/j.bios.2020.112947 PMC7855766

[36] Jin S, Chen H, Pan K, Li R, Ma X, Yuan R, Meng X, He H. State-of-the-art electrochemical biosensors based on covalent organic frameworks and their hybrid materials. Talanta. 2024;270:125557. 38128284 10.1016/j.talanta.2023.125557

[37] Xie LS, Skorupskii G, Dinca M. Electrically conductive metal–organic frameworks. Chem Rev. 2020;120(16):8536–80. 32275412 10.1021/acs.chemrev.9b00766 PMC7453401

[38] Lin Y, Huang Y, Chen X. Recent advances in metal-organic frameworks for biomacromolecule sensing. Chemosensors. 2022;10(10):412.

[39] Muthukumaran MK, Govindaraj M, Kogularasu S, Sriram B, Raja BK, Wang S-F, Chang-Chien G-P. Recent advances in metal-organic frameworks for electrochemical sensing applications. Talanta Open. 2025;11:100396.

[40] Li J-H, Wang Y-S, Chen Y-C, Kung C-W. Metal–organic frameworks toward electrocatalytic applications. Appl Sci. 2019;9(12):2427.

[41] Burnett BJ, Barron PM, Choe W. Recent advances in porphyrinic metal–organic frameworks: materials design, synthetic strategies, and emerging applications. CrystEngComm. 2012;14(11):3839–46.

[42] Chang Y, Lou J, Yang L, Liu M, Xia N, Liu L. Design and application of electrochemical sensors with metal–organic frameworks as the electrode materials or signal tags. Nanomaterials. 2022;12(18):3248. 36145036 10.3390/nano12183248 PMC9506444

[43] Guo X, Lin C, Zhang M, Duan X, Dong X, Sun D, Pan J, You T. 2D/3D copper-based metal-organic frameworks for electrochemical detection of hydrogen peroxide. Front Chem. 2021;9:743637. 34692641 10.3389/fchem.2021.743637 PMC8530376

[44] Leoi MWN, Zheng XT, Yu Y, Gao J, Ong DHS, Koh CZH, Chen P, Yang L. Redefining metal organic frameworks in biosensors: where are we now? ACS Appl Mater Interfaces. 2025;17(9):13246–78. 39984305 10.1021/acsami.4c19307

[45] Kulandaivel S, Chen H-T, Lin C-H, Yeh Y-C. Exploring the potential of iron-based metal–organic frameworks as peroxidase nanozymes for glucose detection with various secondary building units. J Mater Chem B. 2023;11(43):10362–8. 37465898 10.1039/d3tb00981e

[46] Gutiérrez-Serpa A, Pacheco-Fernández I, Pasán J, Pino V. Metal–organic frameworks as key materials for solid-phase microextraction devices—a review. Separations (Basel). 2019;6(4):47.

[47] Howarth AJ, Peters AW, Vermeulen NA, Wang TC, Hupp JT, Farha OK. Best practices for the synthesis, activation, and characterization of metal–organic frameworks. Chem Mater. 2017;29(1):26–39.

[48] Kaushal S, Kaur G, Kaur J, Singh PP. First transition series metal–organic frameworks: synthesis, properties and applications. Mater Adv. 2021;2(22):7308–35.

[49] Vignesh S, Ahmad K, Oh TH. Progress in nickel MOF-based materials for electrochemical biosensor and supercapacitor applications. Biosensors. 2025;15(9):560. 41002300 10.3390/bios15090560 PMC12467741

[50] Buzek D, Demel J, Lang K. Zirconium metal–organic framework UiO-66: stability in an aqueous environment and its relevance for organophosphate degradation. Inorg Chem. 2018;57(22):14290–7. 30371080 10.1021/acs.inorgchem.8b02360

[51] Chattopadhyay K, Mandal M, Maiti DK. A review on zirconium-based metal–organic frameworks: synthetic approaches and biomedical applications. Mater Adv. 2024;5(1):51–67.

[52] Kim JY, Kang J, Cha S, Kim H, Kim D, Kang H, Choi I, Kim M. Stability of Zr-based UiO-66 metal–organic frameworks in basic solutions. Nanomaterials. 2024;14(1):110. 38202565 10.3390/nano14010110 PMC10780619

[53] Chen L, Wang H-F, Li C, Xu Q. Bimetallic metal–organic frameworks and their derivatives. Chem Sci. 2020;11(21):5369–403. 34094065 10.1039/d0sc01432j PMC8159423

[54] Zhang H, Zhang M, Geng Q, Gao X. Co/Ni-MOF as a bifunctional electrode material for electrochemical sensor and oxygen evolution reaction. Mater Lett. 2024;355:135538.

[55] Hang X, Zhao J, Xue Y, Yang R, Pang H. Synergistic effect of Co/Ni bimetallic metal–organic nanostructures for enhanced electrochemical energy storage. J Colloid Interface Sci. 2022;628:389–96. 35932675 10.1016/j.jcis.2022.07.136

[56] Yu K, Zhang J, Hu Y, Wang L, Zhang X, Zhao B. Ni doped Co-MOF-74 synergized with 2D Ti3C2Tx MXene as an efficient electrocatalyst for overall water-splitting. Catalysts. 2024;14(3):184.

[57] Wang D, Le F, Lv J, Yang X, Chen X, Yao H, Jia W. Fe-incorporated nickel-based bimetallic metal–organic frameworks for enhanced electrochemical oxygen evolution. Molecules. 2023;28(11):4366. 37298841 10.3390/molecules28114366 PMC10254281

[58] Liang Z, Chen S, Fan C, Tian G, Zong Z, Wang J, He H, Su H, Guo F. Recent advancements in metal–organic framework-based aptasensors. Dalton Trans. 2025. 10.1039/d4dt02959c. 39660581 10.1039/d4dt02959c

[59] Tolentino MQ, Hartmann AK, Loe DT, Rouge JL. Controlled release of small molecules and proteins from DNA-surfactant stabilized metal organic frameworks. J Mater Chem B. 2020;8(26):5627–35. 32391534 10.1039/d0tb00767f

[60] Evtugyn G, Belyakova S, Porfireva A, Hianik T. Electrochemical aptasensors based on hybrid metal-organic frameworks. Sensors (Basel). 2020;20(23):6963. 33291498 10.3390/s20236963 PMC7729924

[61] Yusuf VF, Malek NI, Kailasa SK. Review on metal–organic framework classification, synthetic approaches, and influencing factors: applications in energy, drug delivery, and wastewater treatment. ACS Omega. 2022;7(49):44507–31. 36530292 10.1021/acsomega.2c05310 PMC9753116

[62] Song Y, Ma S. Pore engineering in metal–organic frameworks and covalent organic frameworks: strategies and applications. Chem Sci. 2025. 10.1039/d5sc01635e. 40535720 10.1039/d5sc01635e PMC12172060

[63] Raptopoulou CP. Metal-organic frameworks: synthetic methods and potential applications. Materials (Basel). 2021;14(2):310. 33435267 10.3390/ma14020310 PMC7826725

[64] Wang S, Chen Y, Wang S, Li P, Mirkin CA, Farha OK. DNA-functionalized metal–organic framework nanoparticles for intracellular delivery of proteins. J Am Chem Soc. 2019;141(6):2215–9. 30669839 10.1021/jacs.8b12705 PMC8212418

[65] Yang J, Zhang Y. Research progress on aptamer electrochemical biosensors based on signal amplification strategy. Sensors (Basel). 2025;25(14):4367. 40732494 10.3390/s25144367 PMC12300297

[66] Tran VA, Doan VD, Le VT, Nguyen T-Q, Don TN, Vien V, Luan NT, Vo GN. Metal–organic frameworks-derived material for electrochemical biosensors: recent applications and prospects. Ind Eng Chem Res. 2023;62(11):4738–53.

[67] Li T, Dong S, Wang E. Label-free colorimetric detection of aqueous mercury ion (Hg2+) using Hg2+-modulated G-quadruplex-based DNAzymes. Anal Chem. 2009;81(6):2144–9. 19227981 10.1021/ac900188y

[68] Zhang DY, Seelig G. Dynamic DNA nanotechnology using strand-displacement reactions. Nat Chem. 2011;3(2):103–13. 21258382 10.1038/nchem.957

[69] Liu H, You Y, Zhu Y, Zheng H. Recent advances in the exonuclease III-assisted target signal amplification strategy for nucleic acid detection. Anal Methods. 2021;13(43):5103–19. 34664562 10.1039/d1ay01275d

[70] Zhao J, Xin M, Cao Y, Yin Y, Shu Y, Ma W. An electrochemical aptasensor for thrombin detection based on the recycling of exonuclease III and double-stranded DNA-templated copper nanoparticles assisted signal amplification. Anal Chim Acta. 2015;860:23–8. 25682243 10.1016/j.aca.2014.12.026

[71] Zhou C, Zhang Y, Huang M, Yang K, Tian J, Lu J. Photoelectrochemical aptasensing for thrombin based on exonuclease III-assisted recycling signal amplification and nanoceria enzymatic strategy. Talanta. 2021;233:122577. 34215069 10.1016/j.talanta.2021.122577

[72] Goda T, Higashi D, Matsumoto A, Hoshi T, Sawaguchi T, Miyahara Y. Dual aptamer-immobilized surfaces for improved affinity through multiple target binding in potentiometric thrombin biosensing. Biosens Bioelectron. 2015;73:174–80. 26067329 10.1016/j.bios.2015.05.067

[73] Gonçalves JM, Martins PR, Rocha DP, Matias TA, Juliao MS, Munoz RA, Angnes L. Recent trends and perspectives in electrochemical sensors based on MOF-derived materials. J Mater Chem C. 2021;9(28):8718–45.

[74] Mourdikoudis S, Dutta S, Kamal S, Gómez‐Graña S, Pastoriza‐Santos I, Wuttke S, Polavarapu L. State‐of‐the‐art, insights, and perspectives for MOFs‐nanocomposites and MOF‐derived (nano) materials. Adv Mater. 2025. 10.1002/adma.202415399. 40255059 10.1002/adma.202415399 PMC12747499

[75] Khosravi A, Zarepour A, Iravani S, Zarrabi A. Recent innovations and developments in MOF-based aptasensors. Microchem J. 2025. 10.1016/j.microc.2025.114954.

[76] Rabiee N, Ahmadi S, Rahimizadeh K, Chen S, Veedu RN. Metallic nanostructure-based aptasensors for robust detection of proteins. Nanoscale Adv. 2024;6(3):747–76. 38298588 10.1039/d3na00765k PMC10825927

[77] Su F, Zhang S, Ji H, Zhao H, Tian J-Y, Liu C-S, Zhang Z, Fang S, Zhu X, Du M. Two-dimensional zirconium-based metal–organic framework nanosheet composites embedded with Au nanoclusters: a highly sensitive electrochemical aptasensor toward detecting cocaine. ACS Sens. 2017;2(7):998–1005. 28750538 10.1021/acssensors.7b00268

[78] Sun D, Luo Z, Lu J, Zhang S, Che T, Chen Z, Zhang L. Electrochemical dual-aptamer-based biosensor for nonenzymatic detection of cardiac troponin I by nanohybrid electrocatalysts labeling combined with DNA nanotetrahedron structure. Biosens Bioelectron. 2019;134:49–56. 30954926 10.1016/j.bios.2019.03.049

[79] Cheng T, Li X, Huang P, Wang H, Wang M, Yang W. Colorimetric and electrochemical (dual) thrombin assay based on the use of a platinum nanoparticle modified metal-organic framework (type Fe-MIL-88) acting as a peroxidase mimic. Microchim Acta. 2019;186(2):94. 10.1007/s00604-018-3209-4 30631938

[80] Li Y, Wang J, Ran Y, Liu Y, Hu X. Dual-mode fluorescence/colorimetric aptasensor based on Zn, Ce-MOF nanocomposite for sensitive detection of isocarbophos. Anal Chim Acta. 2025. 10.1016/j.aca.2025.344739. 41167900 10.1016/j.aca.2025.344739

[81] Li F, Luo M, Tan Y, Liu R, Xu L, Zhao P, Wu Z. A dual-mode electrochemical and fluorescence aptasensor based on CDs@ Cu/Al-MOF and Ti3C2 for the detection of kanamycin in milk. Microchim Acta. 2025;192(8):519. 10.1007/s00604-025-07393-6 40707684

[82] Chen W, Tan Y, Zheng H, Wang Z, Qu Z, Wu C. Advance in metal–organic frameworks hybrids-based biosensors. Microchem J. 2024;206:111441.

[83] Zhang H-T, Zhang J-W, Huang G, Du Z-Y, Jiang H-L. An amine-functionalized metal–organic framework as a sensing platform for DNA detection. Chem Commun. 2014;50(81):12069–72. 10.1039/c4cc05571c 25164253

[84] Lan L, Yao Y, Ping J, Ying Y. Recent progress in nanomaterial-based optical aptamer assay for the detection of food chemical contaminants. ACS Appl Mater Interfaces. 2017;9(28):23287–301. 28632380 10.1021/acsami.7b03937

[85] Cruz-Navarro JA, Hernandez-Garcia F, Romero GAA. Novel applications of metal-organic frameworks (MOFs) as redox-active materials for elaboration of carbon-based electrodes with electroanalytical uses. Coord Chem Rev. 2020;412:213263.

[86] Li C, Zhang H, Liu M, Lang F-F, Pang J, Bu X-H. Recent progress in metal–organic frameworks (MOFs) for electrocatalysis. Industrial Chemistry & Materials. 2023;1(1):9–38.

[87] Qiu W, Wang Q, Yano N, Kataoka Y, Handa M, Gao F, Tanaka H. Flexible flower-like MOF of Cu2 (trans-1, 4-cyclohexanedicarboxylic acid) 2 as the electroactive matrix material for label-free and highly sensitive sensing of thrombin. Electrochim Acta. 2020;353:136611.

[88] Wu H, Li M, Wang Z, Yu H, Han J, Xie G, Chen S. Highly stable Ni-MOF comprising triphenylamine moieties as a high-performance redox indicator for sensitive aptasensor construction. Anal Chim Acta. 2019;1049:74–81. 30612659 10.1016/j.aca.2018.10.022

[89] Cheng T, Li X, Huang P, Wang H, Wang M, Yang W. Colorimetric and electrochemical (dual) thrombin assay based on the use of a platinum nanoparticle modified metal-organic framework (type Fe-MIL-88) acting as a peroxidase mimic. Microchim Acta. 2019;186:1–8. 10.1007/s00604-018-3209-4 30631938

[90] Yu H, Han J, An S, Xie G, Chen S. Ce (III, IV)-MOF electrocatalyst as signal-amplifying tag for sensitive electrochemical aptasensing. Biosens Bioelectron. 2018;109:63–9. 29529509 10.1016/j.bios.2018.03.005

[91] Xue W, Wang Y, Guo L, Zhang H. Zr-MOF functionalized nanochannels: application to regenerative and sensitive electrochemical aptasensing platform. Sens Actuators B Chem. 2023;381:133455.

[92] Jiang J, Cai Q, Deng M. Construction of electrochemical aptamer sensor based on Pt-coordinated titanium-based porphyrin MOF for thrombin detection. Front Chem. 2022;9:812983. 35071191 10.3389/fchem.2021.812983 PMC8776986

[93] Mohammed Ameen SS, Omer KM. Recent advances of bimetallic-metal organic frameworks: preparation, properties, and fluorescence-based biochemical sensing applications. ACS Appl Mater Interfaces. 2024;16(25):31895–921. 38869081 10.1021/acsami.4c06931

[94] Yu H, Xu G, Li L, Peng H, Chang H, Chen Z, Fu H, Chen D, Ji Y, Yu B. Methylene blue-encapsulated Cu (I)/(Ⅱ) mixed-valence MOF with spontaneously recycled catalysis for electrochemical-colorimetric dual-signal readout aptasensor. Microchem J. 2024;205:111202.

[95] Xiong W, Li D, Han Y, Shi H, Huang L, Wei L, Wang B, Zhang Y, Wang T. Enhanced electrochemical detection of thrombin via a dual signal amplification strategy based on Au nanoparticles@ carbon nanosheets and PtCu3 nanoparticles. J Electroanal Chem. 2024;962:118287.

[96] Yang Y, Yang Z, Lv J, Yuan R, Chai Y. Thrombin aptasensor enabled by Pt nanoparticles-functionalized Co-based metal organic frameworks assisted electrochemical signal amplification. Talanta. 2017;169:44–9. 28411820 10.1016/j.talanta.2017.03.037

[97] Zhang Z-H, Duan F-H, Tian J-Y, He J-Y, Yang L-Y, Zhao H, Zhang S, Liu C-S, He L-H, Chen M. Aptamer-embedded zirconium-based metal–organic framework composites prepared by de novo bio-inspired approach with enhanced biosensing for detecting trace analytes. ACS Sens. 2017;2(7):982–9. 28750523 10.1021/acssensors.7b00236

[98] Xie S, Ye J, Yuan Y, Chai Y, Yuan R. A multifunctional hemin@ metal–organic framework and its application to construct an electrochemical aptasensor for thrombin detection. Nanoscale. 2015;7(43):18232–8. 26487089 10.1039/c5nr04532k

[99] Xie F-T, Zhao X-L, Chi K-N, Yang T, Hu R, Yang Y-H. Fe-MOFs as signal probes coupling with DNA tetrahedral nanostructures for construction of ratiometric electrochemical aptasensor. Anal Chim Acta. 2020;1135:123–31. 33070849 10.1016/j.aca.2020.08.007

[100] Yang X, Lv J, Yang Z, Yuan R, Chai Y. A sensitive electrochemical aptasensor for thrombin detection based on electroactive Co-based metal–organic frameworks with target-triggering NESA strategy. Anal Chem. 2017;89(21):11636–40. 29019234 10.1021/acs.analchem.7b03056

[101] Xie P, Wang D, Zhao H, Yin N, Hu S, Qin W, Meng L, Pan X, Yuan Y, Yuan R. Electrochemical biomimetic enzyme cascade amplification combined with target-induced DNA walker for detection of thrombin. Microchim Acta. 2023;190(5):188. 10.1007/s00604-023-05769-0 37079080

[102] Zhang J, Qiang Y, Xu X. An ultrasensitive electrochemical aptasensor for thrombin detection using MoS2 nanoparticles loaded iron-porphyrinic metal-organic framework as signal amplifier. J Electrochem Soc. 2020;167(8):087503.

[103] Ren Q, Xu X, Cao G, Xia J, Wang Z, Liu Q. Electrochemical thrombin aptasensor based on using magnetic nanoparticles and porous carbon prepared by carbonization of a zinc (II)-2-methylimidazole metal-organic framework. Microchim Acta. 2019;186:1–8. 10.1007/s00604-019-3781-2 31471765

[104] Ren Q, Mou J, Guo Y, Wang H, Cao X, Zhang F, Xia J, Wang Z. Simple homogeneous electrochemical target-responsive aptasensor based on aptamer bio-gated and porous carbon nanocontainer derived from ZIF-8. Biosens Bioelectron. 2020;166:112448. 32862844 10.1016/j.bios.2020.112448

[105] Fatah SA, Omer KM. Aptamer-modified MOFs (Aptamer@ MOF) for efficient detection of bacterial pathogens: a review. ACS Appl Mater Interfaces. 2025;17(8):11578–94. 39951394 10.1021/acsami.4c21944

[106] Farzin MA, Naghib SM, Rabiee N. Emerging metal-organic framework (MOF)-based biosensors with high potential for point-of-care determination of biomarkers: mechanisms and applications. TrAC Trends Anal Chem. 2025.

[107] Kidanemariam A, Cho S. Recent advancements in metal–organic framework-based microfluidic chips for biomedical applications. Micromachines. 2025;16(7):736. 40731645 10.3390/mi16070736 PMC12298669

[108] Theyagarajan K, Kim Y-J. Metal organic frameworks based wearable and point-of-care electrochemical sensors for healthcare monitoring. Biosensors. 2024;14(10):492. 39451704 10.3390/bios14100492 PMC11506055

